# Exposure to Metal Mixtures in Association with Cardiovascular Risk Factors and Outcomes: A Scoping Review

**DOI:** 10.3390/toxics10030116

**Published:** 2022-03-01

**Authors:** Gyeyoon Yim, Yuting Wang, Caitlin G. Howe, Megan E. Romano

**Affiliations:** Department of Epidemiology, Geisel School of Medicine at Dartmouth, Lebanon, NH 03756, USA; yuting.wang.gr@dartmouth.edu (Y.W.); caitlin.g.howe@dartmouth.edu (C.G.H.); megan.e.romano@dartmouth.edu (M.E.R.)

**Keywords:** metal mixtures, cardiovascular diseases, blood pressure, hypertension, preeclampsia, dyslipidemia

## Abstract

Since the National Institute of Environmental Health Sciences (NIEHS) declared conducting combined exposure research as a priority area, literature on chemical mixtures has grown dramatically. However, a systematic evaluation of the current literature investigating the impacts of metal mixtures on cardiovascular disease (CVD) risk factors and outcomes has thus far not been performed. This scoping review aims to summarize published epidemiology literature on the cardiotoxicity of exposure to multiple metals. We performed systematic searches of MEDLINE (PubMed), Scopus, and Web of Science to identify peer-reviewed studies employing statistical mixture analysis methods to evaluate the impact of metal mixtures on CVD risk factors and outcomes among nonoccupationally exposed populations. The search was limited to papers published on or after 1998, when the first dedicated funding for mixtures research was granted by NIEHS, through 1 October 2021. Twenty-nine original research studies were identified for review. A notable increase in relevant mixtures publications was observed starting in 2019. The majority of eligible studies were conducted in the United States (*n* = 10) and China (*n* = 9). Sample sizes ranged from 127 to 10,818. Many of the included studies were cross-sectional in design. Four primary focus areas included: (i) blood pressure and/or diagnosis of hypertension (*n* = 15), (ii) risk of preeclampsia (*n* = 3), (iii) dyslipidemia and/or serum lipid markers (*n* = 5), and (iv) CVD outcomes, including stroke incidence or coronary heart disease (*n* = 8). The most frequently investigated metals included cadmium, lead, arsenic, and cobalt, which were typically measured in blood (*n* = 15). The most commonly utilized multipollutant analysis approaches were Bayesian kernel machine regression (BKMR), weighted quantile sum regression (WQSR), and principal component analysis (PCA). To our knowledge, this is the first scoping review to assess exposure to metal mixtures in relation to CVD risk factors and outcomes. Recommendations for future studies evaluating the associations of exposure to metal mixtures with risk of CVDs and related risk factors include extending environmental mixtures epidemiologic studies to populations with wider metals exposure ranges, including other CVD risk factors or outcomes outside hypertension or dyslipidemia, using repeated measurement of metals to detect windows of susceptibility, and further examining the impacts of potential effect modifiers and confounding factors, such as fish and seafood intake.

## 1. Introduction

Cardiovascular diseases (CVDs), a group of heart and vascular diseases, are the leading causes of morbidity and mortality worldwide, contributing to an estimated 18 million deaths each year [1]. While poor nutrition and physical inactivity have been identified as main contributors to CVDs [2], growing evidence also points to the possible roles of environmental metals and metalloids (collectively referred to as “metals” hereafter) in the development of CVDs. Metals persist in the environment [3] and may have detrimental health effects, even at the low concentrations observed in many parts of the world [4]. Sources of exposure for different metals vary, but include drinking water, consuming contaminated food, inhalation of polluted air, and occupational activities (e.g., mining, smelting, and foundries) [3,5]. Co-exposure to more than one metal is common; it is therefore important to understand the potential joint effects of multiple metals on health [5].

Studies using rodent models have demonstrated that exposure to certain metals, such as arsenic (As), cadmium (Cd), chromium (Cr), and lead (Pb), induces heart dysfunction [6] and hypertension [7]. Oxidative stress [8], altered regulation of endocrine and endothelial vascular functions [9,10,11,12], and epigenetic pathways [13,14,15,16] have been identified as possible biological mechanisms. A substantial body of epidemiological evidence has also reported associations between metal exposures and CVD or related outcomes, with three recent reviews summarizing these findings [4,17,18]. However, prior studies have predominantly investigated metals individually.

Humans are simultaneously exposed to thousands of environmental toxicants every day, and these environmental chemicals may be correlated and interact with each other [19]. Recognizing that most individuals are exposed to complex mixtures of chemicals, the National Institute of Environmental Health Sciences (NIEHS) has encouraged the field to move towards systematically addressing the impacts of combined exposures, since its first dedicated funding mechanism for studying mixtures in 1998 [20]. More recently, the NIEHS has held several mixtures workshops and developed the Powering Research through Innovative Methods for Mixtures in Epidemiology (PRIME) Program to enhance understanding of complex exposures, prioritize mixture research goals, and recommend research collaborations across multidisciplinary settings [21,22,23]. As a result, several novel statistical methods have been developed for evaluating the effects of environmental chemical mixtures on health outcomes, including Bayesian kernel machine regression (BKMR) [24], weighted quantile sum regression (WQSR) [25], and quantile-based g-computation [26]. However, to our knowledge, no study has yet reviewed epidemiologic studies applying these approaches to investigate how metals impact cardiovascular risk factors and outcomes in the context of multipollutant exposures.

We therefore present a scoping review of the emerging evidence for cardiovascular impacts of metal mixtures. Our objectives were to describe key gaps in our current scientific understanding of metal mixture impacts on cardiovascular risk factors and outcomes and to guide future investigators as they design and conduct studies on this topic or refine topics for future systematic reviews. We describe recent studies in this area, synthesize common themes, and suggest future directions for metal mixtures research.

## 2. Methods

### 2.1. Search Strategy

This review was performed in accordance to the Preferred Reporting Items for Systematic Reviews and Meta-Analyses Extension for Scoping Reviews (PRISMA-ScR) [27]. The protocol was prepared a priori and registered with the Open Science Framework [28].

To identify observational studies that examined the associations of exposure to metal mixtures with cardiovascular risk factors and outcomes applying multipollutant approaches, we developed a comprehensive search strategy with assistance from an experienced librarian. The following databases were searched: MEDLINE (PubMed), Scopus, and Web of Science. The search strategy was developed primarily for MEDLINE (PubMed), with adapted searches for the other databases. A detailed description of the search strategy can be found in Table S1. A literature search was conducted in each database, restricting to papers published between 1998, which is when the NIEHS first funded mixtures grants [20], through 1 October 2021. No language restriction was imposed. Metals were included as specific text-word terms in our search to enhance sensitivity, adding Medical Subject Headings (MeSH) for trace elements. Key search terms for the outcomes were also developed using both MeSH terms to compile synonyms for cardiovascular risk factors and outcomes, such as “cardiometabolic risk factors” [Mesh] or “vascular diseases” [Mesh], and more specific text-word terms to increase sensitivity, including stroke, dyslipidemia, and preeclampsia. We further included the commonly used mixture analysis methods that were introduced by the NIEHS, such as BKMR and WQSR [29]. The search strategies were reviewed by other experienced researchers (M.E.R. and C.G.H.) prior to execution. We also manually checked the reference lists of the eligible research to identify additional pertinent studies.

### 2.2. Study Selection

The search records were pooled in Endnote 20 (Clarivate, Philadelphia, PA, USA), and literature screening was carried out using Rayaan (available online: https://www.rayyan.ai/ (accessed on 15 October 2021)). We used the Populations, Exposures, Comparators, and Outcomes (PECO) framework to formulate eligibility criteria (Table 1) [30]. After de-duplication performed in Endnote, titles and abstracts were screened for relevance (G.Y.). For the studies deemed potentially eligible, at least two of the four investigators (C.G.H., M.E.R., Y.W., and G.Y.) independently evaluated the full-text papers for studies that had been pre-screened to determine eligibility. Studies were included if they fulfilled the following prespecified eligibility criteria: the study: (1) was conducted in humans; (2) was an original research study published in a peer-reviewed journal; (3) used a cohort, cross-sectional, case-control, or panel study design; (4) evaluated at least three or more metals, thus meeting the NIEHS definition of a mixture [31]; (5) measured metals at the individual level, quantified in human biological samples; and (6) used a multipollutant approach rather than traditional regression. Papers were excluded if: (1) they did not describe an original research study (e.g., review, editorial, case report, case series, or non-research letter); (2) the study was experimental or used an ecologic study design; (3) the study focused on prediction models, rather than hypothesis-driven approaches; (4) the unit of analysis was not a study participant (e.g., studies based on spatially interpolated data); (5) metal exposures were estimated using surrogate measures (e.g., by diet, job title, or proximity to contamination sources); (6) the study population was occupationally exposed to metals; (7) cardiovascular risk factors or outcomes were not measured; or (8) the primary outcomes were cardiovascular specific mortality or hospital admission outcomes (due to the possibility of including competing risks [32]). In a few cases, more than one publication from the same cohort/study population was identified; these studies were considered eligible for inclusion if different metal combinations, cardiovascular risk factors/outcomes, or mixtures analysis methods were used. Any disagreements among the four screeners about study eligibility were resolved by consensus discussion.

### 2.3. Data Extraction

Data on the following characteristics were extracted from the included studies into a premade Google Sheets form: first author, year of publication, study location, study design, sample size, metals evaluated, exposure biomarkers, concentration of metals measured in biospecimens (i.e., range, median, or mean), outcomes evaluated, mixture analysis method(s) applied, covariates, and a summary of the study’s main findings (Table 2). Some mixture analysis methods provide quantitative risk estimates (e.g., WQSR), but not all do (e.g., regression trees). Where a quantitative effects estimate was available, we report it in our summary table, but qualitative descriptions of associations were necessary for some methods. Four investigators (C.G.H., M.E.R., Y.W., and G.Y.) independently performed data extraction. Two investigators were assigned to each paper, and discrepancies in extracted information were resolved in discussion with a third reviewer. As a scoping review, we did not include a quality evaluation of the included studies [33,34].

### 2.4. Data Synthesis and Analysis

We reviewed and synthesized the evidence separately for each cardiovascular risk factor or outcome. We were not able to perform a meta-analysis, because no method currently exists that can quantitatively evaluate results from studies using different multipollutant approaches. Instead, all eligible studies were summarized narratively.

## 3. Results

**Figure 1:**
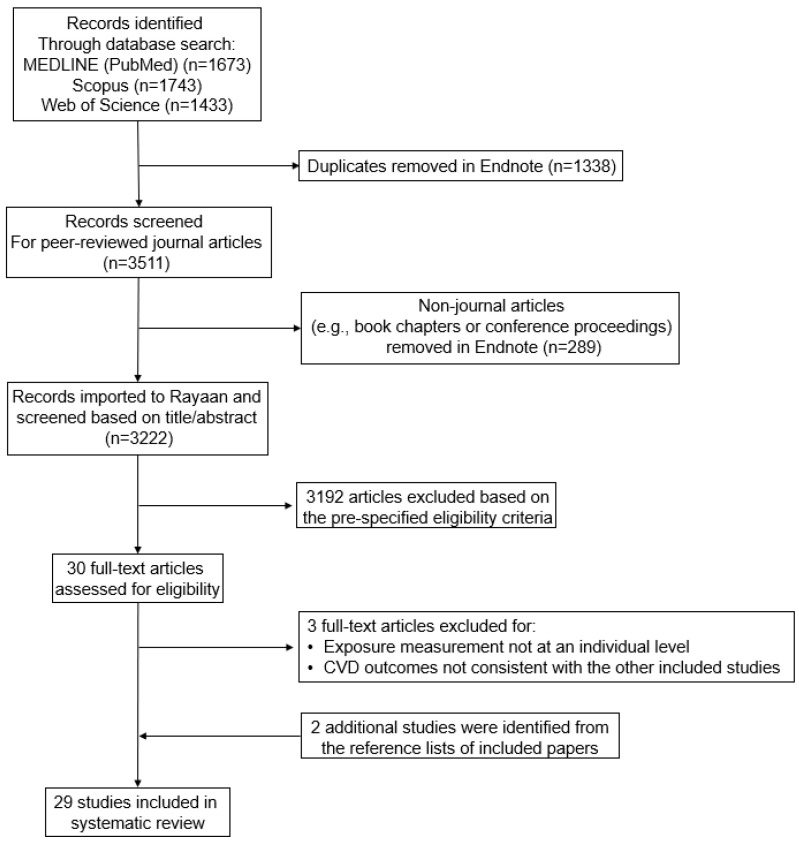
Flow chart of literature review and study selection for papers published between 1998 through 1 October 2021.

We identified 1673 studies with MEDLINE (PubMed), 1743 with Scopus, and 1433 with Web of Science (Figure 1). Of these, a total of 3511 citations reflected unique studies. After removing studies that were not peer reviewed, such as book chapters and conference proceedings, 3222 studies were retained for title/abstract screening. The PECO criteria were used to guide title and abstract screening, and 3192 studies were excluded, leaving 30 for full text screening. Of these, three studies were excluded because the metal exposures were not measured at the individual level [35,36] or definitions for the cardiovascular risk factors or outcomes were not consistent with the other studies included [37]; two additional studies [38,39] were identified via manual search or reference lists of the included studies. 

We also found two potentially eligible studies but did not include them in this review because mixed-effects regression models, not multipollutant approaches considered in this review, were used among the study population stratified by baseline metal concentrations to estimate joint associations of metal mixtures with blood and pulse pressure trajectories [40] or the clusters identified from a multi-pollutant approach were not linked to CVD-related outcomes [41]. Twenty-nine studies were selected for further review, which were all written in English.

**Figure 2:**
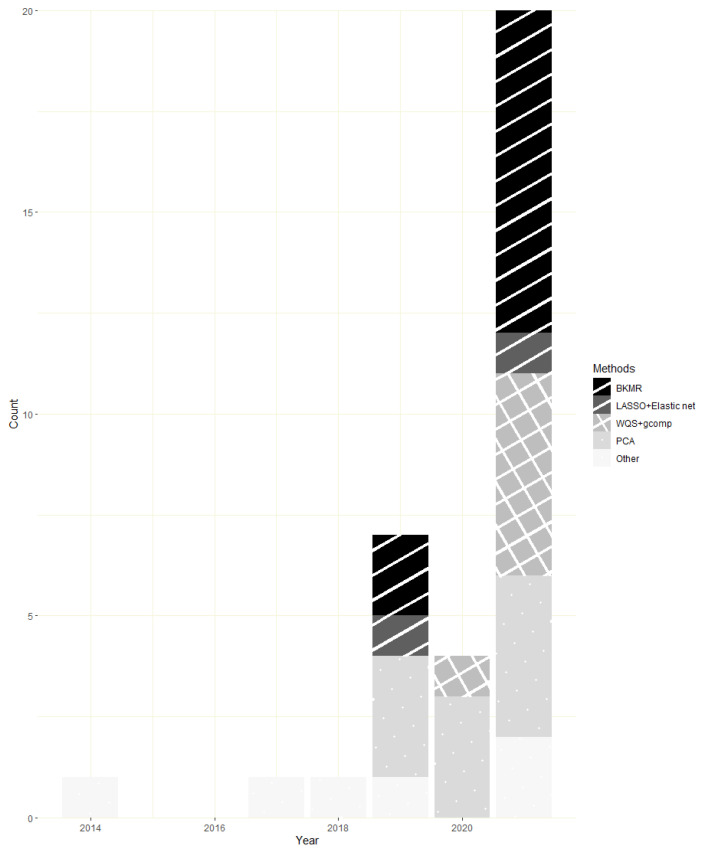
Number of included studies published by year and multi-pollutant approach. Note: Some studies included multiple cardiovascular disease (CVD) related outcomes or mixture analysis methods.

Of the 29 studies included in our review, most (*n* = 26) were published between 2019 and 2021, with the number of studies investigating the association of metal mixtures exposure with CVD risk factors and outcomes doubling in 2021 (Figure 2). Ten studies were conducted in the USA, followed by 9 in China (Table 2). While 6 out of the 10 USA studies were based on NHANES data [42,43,44,45,46,47], none were excluded as they focused on unique combinations of metal components, evaluated different CVD-related outcomes, or used different mixtures analysis methods. Sample sizes ranged between 127 (a panel study of adults in China [48]) to 10,818 (a cross-sectional study conducted in the USA NHANES [43]).

The majority of studies were either cross-sectional in design (*n* = 13) [39,42,43,44,45,46,47,49,50,51,52,53,54] or prospective cohort studies (*n* = 10) [38,55,56,57,58,59,60,61,62,63]. Cardiovascular outcomes and risk factors were categorized into four groups: (i) blood pressure (BP) and hypertension, (ii) preeclampsia, (iii) dyslipidemia and serum lipid markers, and (iv) clinical CVD outcomes, including stroke, coronary heart disease (CHD), and myocardial infarction (MI). Blood pressure and hypertension were the most commonly studied outcomes (*n* = 15) [38,39,42,44,45,46,47,49,50,51,55,56,57,58,59], followed by dyslipidemia and serum or blood lipid markers (*n* = 5) [43,52,53,55,60], and preeclampsia (*n* = 3) [61,62,64]. Other CVD outcomes were investigated less frequently [48,54,55,63,65,66,67,68].

**Figure 3:**
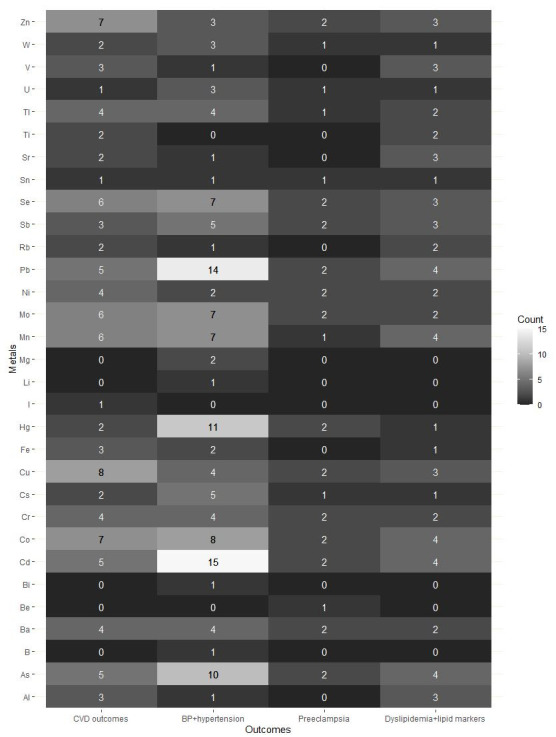
Heat map of metals and cardiovascular disease (CVD) risk factors or outcomes in the included studies. Numbers represent the number of reviewed publications corresponding to each metal (columns) and CVD risk factors or outcomes (rows). Note: The sum of the numbers presented here exceed the total number of selected studies (*n* = 29), as each study included a minimum of three metals within their mixtures. Some studies also evaluated more than one CVD-related outcome or risk factor, including stroke, coronary heart disease, and myocardial infarction; blood pressure; diagnosis of hypertension, preeclampsia, and dyslipidemia; and levels of lipid serum or blood lipid markers.

The frequency by which specific metals have been evaluated is shown separately by CVD risk factors or outcomes in Figure 3. Metal components from each included study were pooled out, rearranged, and counted by the four CVD-related outcomes. Among the included studies, a total of 31 different metals were studied in relation to CVD risk factors or outcomes. The most commonly studied metal was Cd, followed by Pb, mercury (Hg), and As. While most of the retrieved studies included both toxic metals and essential elements in their mixtures, five studies [43,44,46,49,64] only included toxic metals in their analysis, with one study [67] focusing on essential elements only. Overall, the results were inconsistent within and across the different endpoints. Possible reasons for these discrepancies include differences in population demographics, exposure patterns and levels, mixture components, study designs, biospecimens measured, the timing of exposure and outcome assessment, population-specific unmeasured confounding, CVD outcome definitions, and the statistical approaches used [69].

Blood was most commonly used for metals assessment (*n* = 15), followed by both blood and urine (*n* = 7), urine (*n* = 6), and toenail clippings (*n* = 1). The majority of studies quantified metal concentrations using inductively coupled plasma-mass spectrometry (ICP-MS), although one study [59] used inductively coupled plasma optical emission spectrometry and one study [55] used inductively coupled plasma triple quadrupole mass spectrometry (ICP-QQQ). Metal concentrations for each of the 29 studies are shown in Table S2. The most frequently employed mixture analysis methods included BKMR, WQSR, and principal component analysis (PCA). Most of the studies adjusted or matched for key confounding factors, such as age, sex and smoking status, in their analyses [17].

### 3.1. Blood Pressure and Hypertension

A total of 15 studies evaluated metal mixtures in relation to blood pressure and risk of hypertension [38,39,42,44,45,46,47,49,50,51,55,56,57,58,59]. Almost half of these studies (*n* = 7) were conducted in the USA [42,44,45,46,47,50,58]. The remaining studies were conducted in Bangladesh [57], Canada [51], China [59], Greece [56], Mexico [55], South Korea [49], Spain [39], or across Europe [38].

Almost half (*n* = 7) of these studies focused on child or adolescent populations, of which four studies focused on metal mixture exposures during the prenatal period. Sample sizes ranged from 133 to 10,566 participants. Most of the studies were cross-sectional in design [39,42,44,45,46,47,49,50,51], and the remaining studies were cohort studies [38,55,56,57,58,59]. Metal concentrations were most frequently measured in blood (*n* = 6), followed by both blood and urine (*n* = 5), urine (*n* = 3), and toenail clippings (*n* = 1). Procedures for blood pressure measurement and hypertension definitions were consistent across the included studies. However, diverse multipollutant approaches were employed, including PCA, BKMR, WQSR, quantile-based g-computation, k-medoids, regression tree, environmental risk score (ERS), and deletion-substitution-addition (DSA) algorithm.

Six studies meeting our inclusion criteria, all conducted in adults, have investigated impacts of metal mixtures on hypertension. Of these, only one prospective study by Zhong et al. [59] has investigated metal mixture impacts on hypertension. This study was conducted in the Wanjiang Cohort, which follows adults living along the Yangtze River in China. A large panel of metals, measured in urine (aluminum [Al], As, boron [B], barium [Ba], bismuth [Bi], Cd, cobalt [Co], Cr, copper [Cu], iron [Fe], Hg, lithium [Li], magnesium [Mg], manganese [Mn], molybdenum [Mo], nickel [Ni], Pb, rubidium [Rb], selenium [Se], strontium [Sr], titanium [Ti], and zinc [Zn]), were examined in relation to hypertension using a 2-stage approach. First, single-metal analyses were conducted using traditional linear regression. Hierarchical BKMR was then used to simultaneously evaluate metals that were found to be associated significantly with hypertension in step 1 as a mixture. Metals were grouped based on their pairwise correlations and knowledge of common exposure sources. BKMR identified a positive association between Cd and hypertension, and similar trends were observed for Mo and Zn. A cumulative increase in all metals was also associated with greater odds of hypertension. Additionally, a potential interaction between Cd and Zn was detected in relation to risk of hypertension. Park et al. [42] conducted a cross-sectional analysis of metal mixtures, measured in both blood and urine, and hypertension among adults in the USA NHANES. An ERS was constructed as a summary measure to investigate the multipollutant impact of a large panel of metals (antimony [Sb], As, Ba, Cd, Co, cesium [Cs], Mo, Hg, Pb, thallium [Tl], tungsten [W], and uranium [U] in urine; Cd, Hg, and Pb in whole blood; and arsenobetaine, monomethyl arsenic, and dimethyl arsenic in urine) on gamma-glutamyl transferase (GGT) using adaptive elastic-net with main effects and pairwise interactions (AENET-I), Bayesian additive regression tree (BART), BKMR, and Super Learner. In the second step, associations between the GGT-ERS and cardiovascular endpoints, including hypertension, were evaluated. The authors concluded that the ERS based on AENET performed better than the other approaches in terms of prediction performance. The ERS included Cd (urine), dimethylarsonic acid, monomethylarsonic acid, Co, and Ba as significant predictors of GGT, and all ERS showed significant associations with risk of hypertension, with a one standard deviation (SD) increase in ERS from AENET-I being associated with a 1.26 (95% CI: 1.15–1.38) higher odds of hypertension. Wang et al. [45] similarly examined the association between exposure to the same set of metals that Park et al. evaluated [42] and hypertension among the USA NHANES participants using a 2-stage approach. In step 1, AENET was used to identify which elements from the mixture [42] were associated with waist circumference. While both studies constructed ERS using AENET, Wang et al. additionally included squared terms and pairwise interactions in their model. In step 2, an ERS was constructed from the metals selected in step 1, including Pb, Cd, Hg, Ba, and Tl, which was then examined in relation to hypertension. The 90th versus 10th percentile of the ERS was associated with a 1.55 (95% CI: 1.28–1.88) higher odds of hypertension. Another cross-sectional study by Xu et al. [50] utilized data from the Gulf Long-Term Follow-Up Study in the USA, a cohort of individuals who participated in cleanup efforts for the Deepwater Horizon oil spill and individuals who completed safety trainings but were not hired. Whole blood measures of multiple metals (Cd, Hg, Mn, Pb, and Se) were investigated simultaneously in relation to hypertension using quantile-based g-computation, but results were null (OR: 0.96, 95% CI: 0.73–1.27 for a quartile increase in the metal mixture). In a cross-sectional analysis of adults in the Korean NHANES, Kim and Park [49] examined the combined impact of three metals (Cd, Hg, and Pb) measured in whole blood on hypertension and prehypertension using WQSR. A quartile increase in the WQS index was associated with greater odds of hypertension (odds ratio [OR]:1.29, 95% confidence interval [CI]: 1.19–1.40), with Pb contributing the most to this association.

Of the six studies which evaluated metals in relation to risk of hypertension, only one by Zuk et al. [51] considered other chemicals as part of the mixture. The authors used PCA to investigate how exposure to multiple classes of environmental contaminants were associated with risk of hypertension among indigenous adults residing in Canada. The environmental chemicals mainly consisted of persistent organic chemicals (nine polychlorinated biphenyls [PCBs] and seven organochlorine pesticides or metabolites), along with four metals (Cd, Hg, Pb, and Se) measured in blood. The top two principal components (PCs) had high loadings for all PCBs, other organic compound concentrations (OCs), and Hg (PC-1), or Pb (PC-2). In the modified Poisson regression model adjusting for these two PCs as independent predictors for risk of hypertension, PC-1 was associated with higher risk of hypertension (adjusted prevalence ratios [aPR]: 1.08; 95% CI: 1.003–1.172).

Two studies examined metal mixture impacts on blood pressure (BP) in adults. Everson et al. [47] used regression trees to identify predictors for systolic blood pressure (SBP) and diastolic blood pressure (DBP) from nine metals (Ba, Cd, Co, Cs, Mo, Sb, Tl, W measured in urine, Pb measured in whole blood) among those below the age of 60 in the USA NHANES. They found that higher concentrations of Sb, Cd, W, and Pb predicted higher SBP, while Cs and Mo predicted lower SBP and DBP. Predictors varied by race/ethnicity, as Cd was the main predictor for SBP among non-Hispanic black adults, whereas the combination of high W and high Sb was the main predictors for SBP among non-Hispanic white adults. Similarly, Yao et al. [44] assessed metal mixtures impacts on BP in the USA NHANES using a two-step approach. In the first step, they applied k-medoids clustering to categorize the study population into two subgroups based on either urine levels of Pb, Cd, and total As, or blood levels of Pb, Cd, and Hg. Secondly, linear regression models were used to examine the association between the exposure subgroups and BP. The “high-exposure” group based on blood levels was significantly associated with increased SBP and DBP compared with the “low-exposure” group, and the “high-exposure” group based on urine levels was significantly associated with increased DBP, but not SBP.

Four prospective cohort studies investigated the impacts of prenatal exposure to metal mixtures on childhood BP. Zhang et al. [58] examined the relationship between exposure to five metals (Pb, Hg, Cd, Se, and Mn in maternal whole blood samples collected 24 to 72 h after delivery) and childhood BP between 3 and 15 years of age from the Boston Birth Cohort. Overall, metal mixtures were not associated with BP. BKMR with hierarchical variable selection identified essential elements as having stronger associations with SBP than toxic metals. Among essential elements and toxic metals, Se and Pb, respectively, had the largest conditional posterior inclusion probabilities (PIPs), which quantify the relative importance of each component within the mixture. BKMR also identified a potential interaction between Mn and Cd, indicating that the inverse association between Mn and childhood SBP was strong at higher concentrations of Cd. Howe et al. [56] assessed the association between prenatal exposure to metal mixtures (Mg, Co, Se, Mo, As, Cd, Sb, and Pb measured in maternal urine samples collected in early pregnancy) and elevated BP at 11 years of age in the Rhea cohort in Greece using BKMR. They also used Bayesian Varying coefficient kernel machine regression (BVCKMR) to assess the impact of this mixture on BP trajectories from 4–11 years of age. A J-shaped association was found between Mo and Co exposure with both SBP and DBP at baseline (age 4). Cd was inversely associated with DBP but not with SBP at baseline. From ages 4 to 11, however, Mo was associated with lower per-year increases in DBP, and Co was associated with lower per-year increases in both SBP and DBP. Mg was not associated with BP at baseline but was associated with higher per-year increases in both SBP and DBP from ages 4 to 11. Mo and Pb showed a J-shaped association with BP at age 11, and a possible synergistic interaction was identified between Mo and Pb and BP at ages 4 and 11. Kupsco et al. [55] evaluated the association between prenatal exposure to eleven metals (As, Cd, Co, Cr, Cs, Cu, Mn, Pb, Sb, Se, and Zn measured in maternal whole blood samples collected during the second trimester) and BP among children 4–6 years old in the Mexico Programming Research in Obesity, Growth Environment and Social Stress (PROGRESS) birth study. BKMR indicated that the joint and individual effect of metals on BP were close to null. Warembourg et al. [38] conducted an exposome-wide association study (ExWAS) using DSA to study associations between the prenatal and postnatal exposomes and childhood BP at 6–11 years of age using pooled data from 6 longitudinal-based European birth cohorts. The prenatal exposomes included 10 metals As, Cd, Co, Cs, Cu, Hg, Mn, Mo, Pb, and Tl measured in maternal whole blood samples collected during pregnancy. The postnatal exposomes included the same 10 metals measured in child blood samples collected at 6–11 years of age. The prenatal and postnatal exposomes were investigated separately in relation to child BP. Postnatal Cu was associated with increased DBP at 6–11 years of age, but none of the prenatal metal exposures were selected as important predictors of child BP.

Desai et al. [46] used BKMR to evaluate associations between metal mixtures (Pb and Hg in whole blood; Cd and total As in urine) on BP among children and adolescents between 8 and 17 years of age in the USA NHANES. BKMR identified inverse associations between low-level Pb, Hg, As, and Cd and DBP. A suggestive inverse association was also identified between Cd and both SBP and DBP. Notably, urinary Cd concentrations (median: 0.06 μg/g creatinine) among this NHANES sample of children and adolescents were low, with 37% of Cd measures in this population falling below the LOD (limit of detection). Castiello et al. [39] used PCA to determine the impacts of metal mixtures (As, Cd, Hg, Ni, Pb, Mn, and Cr in urine) on BP among Spanish male adolescents 15–17 years of age. PC-1 had high loadings from Ni, Cr, and Mn; PC-2 had high loadings for As and Hg; and PC-3 had high loadings for Cd and Pb. A positive association was found between PC-2 and SBP. Shih et al. [57] used three mixture approaches (BKMR, PCA, and WQS) to evaluate the association of seventeen metals (Al, As, Cd, Cr, Co, Cu, Fe, Pb, Mn, Hg, Mo, Ni, Se, tin [Sn], U, vanadium [V], and Zn measured in child’s toenail) with BP at 5–7 years of age in Bangladesh. BKMR identified an overall negative association between the metal mixture and SBP. Of all metals, Sn had the largest PIP for SBP and Se, Mo and Hg had the highest PIPs for DBP. Cr was positively associated with SBP and DBP among boys but not girls, whereas Cu was negatively associated with DBP among girls but not boys. Most relationships were linear, except for the associations of Sn with SBP, and Cu and Se with DBP in girls. The WQS positive index was associated with increased DBP in the whole study population (β coefficient: 1.07, 95% CI: 0.09–2.05) and also among boys (β coefficient: 1.68, 95% CI: 0.21–3.14). Se, Ni and Zn contributed the most to the positive index for DBP in the overall population while Mo, Se, Ni, Cd, Pb and As contributed most to the index among boys. The authors also used PCA, focusing on relationships between the top three PCs and BP. Among boys, PC-2 (highly loaded with Cu, Zn, Se, Cd and Pb) was associated with higher DBP, and PC-3 (highly loaded with Hg) was associated with lower DBP. Among girls, Sn, Se and Zn had higher loadings for PC3, which was associated with a lower SBP.

### 3.2. Preeclampsia

Three studies based in the USA [61,62] and China [64] investigated metal mixture impacts on risk of preeclampsia or changes in blood pressure during pregnancy [61,62,64]. The sample sizes ranged from 383 to 1688. Of these, two studies were prospective cohort studies [61,62] and the remaining study was case-controlled in design [64]. No metal was included in all three studies, but As, Cd, Co, Cr, Hg, Ni, Pb, and Sb were each included in two of the three studies. Liu et al. [62] assessed the association between exposure to 8 metals (Ba, Cs, Sb, Co, Cu, Mo, Se, and Zn) measured in first trimester blood samples and BP at baseline as well as BP changes during pregnancy. Study participants were drawn from the Eunice Kennedy Shriver National Institute of Child Health and Human Development Fetal Growth Studies-Singleton cohort in the USA. Using BKMR, a joint association was observed between the metal mixture and both SBP and DBP at baseline. Individual metals, accounting for the rest of the mixture, were also evaluated; each interquartile range (IQR) increase in Cu was associated with a 0.67 mmHg (95% CI: 0.02–1.32) higher SBP and a 0.60 mmHg (95% CI: 0.08–1.12) higher DBP at baseline. Similarly, each IQR increment in Se was associated with a 0.67 mmHg higher SBP (95% CI: 0.05–1.29). The study by Bommarito et al. [61] included pregnant women enrolled in the LIFECODES birth cohort in Boston who were originally selected for a case-control study of preterm birth; inverse probability weighting was used to account for the disproportionate number of preterm births in their sample. Metal mixture impacts on preeclampsia were examined using PCA, focusing on 11 metals measured in third trimester maternal urine samples that were above detection for >30% of participants: As, beryllium [Be], Cd, Cu, Hg, Mn, Ni, Pb, Se, Sn, Tl, and Zn. The top three metal PCs were not significantly associated with preeclampsia, and no significant interactions were identified between the PCs. However, individuals with higher scores for PC-2 (characterized by high loadings for Cd, Mn, and Pb) who had scores below the median for PC-1 (characterized by high loadings for Cu, Se, and Zn) had an increased risk of developing preeclampsia. Wang et al. [64] evaluated associations between eight metals (Cr, Co, Pb, Hg, Sb, Cd, As, and Ni), measured in maternal whole blood collected at delivery, and preeclampsia using two mixture modeling approaches: WQSR and PCA. With WQSR, they found that a tertile increase in the weighted sum of metals was associated with higher odds of developing preeclampsia (OR: 1.68, 95% CI: 1.20–3.33), with Cr contributing the most to this association. In the PCA analysis, individuals with scores in the highest tertile for PC-2 (characterized by high loadings for Cr and As) had an increased odds of preeclampsia (OR: 1.59, 95% CI: 1.10–2.31) compared with individuals with scores in the lowest tertile. Furthermore, individuals with scores in the highest and middle tertiles for PC-3 (characterized by high loadings for Pb and Hg) had an increased odds of early onset preeclampsia (gestational age <34 weeks) compared to individuals with scores in the lowest tertile (OR: 2.19, 95% CI: 1.34–3.60 and OR: 2.48, 95% CI: 1.45–4.25 comparing the middle and high tertiles with the lowest tertile, respectively). In stratified analyses, the positive association between PC-2 and preeclampsia was stronger among women who were overweight or obese. Both Bommarito et al. and Wang et al. used the same definition of preeclampsia.

### 3.3. Dyslipidemia and Lipid Markers

Five peer-reviewed papers which met our inclusion criteria examined associations between metal mixture exposures and risk of dyslipidemia [52,60] or lipid markers, such as triglyceride (TG), total cholesterol (TC), high-density lipoprotein cholesterol (HDL-C), low-density lipoprotein cholesterol (LDL-C), and leptin levels [43,53,55,60]. These studies were conducted in China [52,53,60], the USA [43], and Mexico [55] with sample sizes ranging from 461 to 10,818 participants. Two of these were cohort studies, and three were cross-sectional in design. The investigators used a range of methods to evaluate the impacts of metal mixtures on dyslipidemia, including PCA, ERS, WQSR, and BKMR.

Jiang et al. [60] and Zhu et al. [52] investigated the associations of exposure to multiple metals with risk of dyslipidemia. Both studies: (i) used the same definition of dyslipidemia, (ii) measured metal concentrations in blood samples, (iii) employed a combination of PCA and logistic regression, and (iv) adjusted for a similar set of covariates. However, the two studies differed by participant age distribution and study design. Jiang et al. [60] measured a large panel of metals (Al, Sb, As, Ba, Co, Cu, Pb, Mn, Mo, Ni, Rb, Se, Sr, Tl, Ti, V, and Zn) in plasma among participants drawn from prior studies nested within the Dongfeng-Tongji cohort in China. Participants were prospectively followed for an average of 5 years to ascertain incident dyslipidemia using plasma measurements of TC, TG, HDL-C, and LDL-C. PCA results suggested that individuals with scores in the highest quartile of PC-1 (which had high loadings for plasma Al, As, Ba, Pb, V, and Zn) had a 40% increase in risk of dyslipidemia (OR: 1.40; 95% CI: 1.07–1.84) compared with individuals with scores in the lowest quartile. No significant associations were identified for the other four PCs evaluated and dyslipidemia. Zhu et al. used a cross-sectional design and focused on adults >60 years old in China [52]. They used PCA to reduce the dimensionality of seven metals (Sr, Cd, Pb, V, Al, Co, and Mn) measured in blood. Combined exposure to Al, Co, and V was associated with reduced risk for dyslipidemia, whereas Cd, Sr, and Pb were associated with an increased risk.

Jiang et al. [60], Li et al. [53], and Park et al. [43] reported relationships between exposure to metal mixtures and concentrations of lipid markers in adult populations. Metals measured in at least two out of these three studies included Al, Ba, Cd, Co, Cu, Mn, Mo, Ni, Pb, Rb, Sb, Se, Sr, Ti, Tl, V, and Zn; As was evaluated in all three studies. Utilizing a combination of PCA and logistic regression, Jiang et al. [60] reported positive associations between PC-1 (characterized by high loadings for Al, As, Ba, Pb, V, and Zn) and risk of low HDL-C and high LDL-C, and between PC-3 (characterized by high loadings for Cu, Rb, and Se) and risk of high TC and low HDL-C. Li et al. [53] conducted a cross-sectional study among healthy adults living in the Shimen and Huayuan Counties of the Hunan Province in China to investigate the influence of toxic metals, measured in urine, and micronutrient metals, measured in plasma, on blood lipids (TG, TC, HDL-C and LDL-C) using a combination of WQSR and BKMR-based approaches. Among participants in the Huayuan area, a grouped WQSR analysis identified positive associations between essential metals (Co, Cr, Cu, Fe, Mn, Mo, Ni, Se, Sn, Sr, V, and Zn) and higher concentrations of TG, TC, and LDL-C and an inverse association between toxic metals (Al, As, Ba, Cd, Rb, Sb, Ti, Tl, U, and W) and TG and TC. In contrast, BKMR models did not provide strong evidence of joint effects. However, the BKMR analysis identified associations for both Ti and Zn with TG levels in both study sites; associations for other metals varied by site. Nonlinear associations with total cholesterol and LDL-C were also observed for certain metals, including an inverse U-shaped relation between plasma Fe and LDL-C in the Huayuan area. Moreover, a possible interaction between Zn and Cd was observed in relation to LDL-C in the Huayuan area. Using data from the USA NHANES, Park et al. [43] developed an ERS incorporating measurements of 134 environmental toxicants, including blood concentrations of Pb, Cd, and total Hg and urinary concentrations of Cd, Hg, and both total and speciated As. The ERS was constructed as a weighted sum of the exposures that were selected using regression analysis, followed by logistic regression models with dichotomized each blood lipid measure (TC, HDL, LDL, and TG). Among the pollutants identified as important predictors of blood lipids, urinary Sb was associated with elevated HDL (>40 mg/dL for men and >50 mg/dL for women), whereas the associations for urinary Cd and blood Pb were not significant. In contrast, blood Pb showed a significant positive association with the odds of elevated LDL (>130 mg/dL). Although urinary Cd was also positively associated with the odds of elevated LDL, the association was weak and was not statistically significant.

Of the selected studies, Kupsco et al. [55] was the only study that investigated the association between prenatal exposure to metal mixtures and the concentrations of lipid markers among children. While a higher level of Se was associated with lower TG, higher concentrations of Sb and As were associated with lower leptin levels. Kupsco et al. did not identify evidence for interactions within the metal mixture or non-linear relationships.

### 3.4. Additional CVD Outcomes

We identified eight studies which evaluated the relationship between exposure to metal mixtures and other CVD outcomes [48,54,55,63,65,66,67,68]. Diverse CVD outcomes were examined across studies, including stroke [63,65,66,67], incident CHD [63], incident MI [67], carotid intima-media thickness [54], aortic dissection (AD) [68], arterial stiffness of peripheral arteries [48], and cardiometabolic component scores [55]. Half of these studies (*n* = 4) were conducted in China [48,65,66,68], with the remaining studies performed in Canada [54], Germany [67], Mexico [55], and Spain [63]. A range of study designs, including case-control (*n* = 3), cohort (*n* = 2), case-cohort (*n* = 1), cross-sectional (*n* = 1), and panel studies (*n* = 1) were used for these investigations. Sample sizes ranged between 127 to 2554 participants. Most studies (*n* = 6) measured metal concentrations in blood, while two measured metal concentrations in urine. A wide range of multipollutant approaches were employed across these studies, including PCA, BKMR, WQSR, Elastic net, and least absolute shrinkage and selection operator (LASSO).

Cabral et al. [67], Domingo-Relloso et al. [63], Wen et al. [65], and Xiao et al. [66] reported the associations between metal mixture exposures and stroke. Across these four studies, Cu and Zn were evaluated most commonly, followed by Co, Mn, Mo, and Se. Findings from Cabral et al. [67] employed PCA to assess risk of incident CVD outcomes (MI and stroke) by co-exposure to six essential elements (Mn, Fe, Cu, Zn, iodine [I], and Se) measured in blood among 2087 adults who participated in the European Prospective Investigation into Cancer and Nutrition (EPIC)-Potsdam cohort study. PC-1 was characterized by high loadings for Mn, Fe, and Zn, whereas PC-2 was characterized by high loadings for Cu, I, and Se. PC-2 was associated with an increased risk of developing incident MI or stroke. Domingo-Relloso et al. [63] utilized data from 1171 adults in the Hortega Follow-Up Study in Spain. A mixture of nine metals (Sb, Ba, Cd, Cr, Co, Cu, Mo, V, and Zn) measured in urine were evaluated using a probit extension of BKMR; accounting for the mixture, Cu, Zn, Sb, Cd, Sr, and V were associated with an increased risk of cardiovascular (CHD and stroke) incidence. The study by Wen et al. [65] used a case-control design and included 2554 adults from Shenzhen, China, measuring 11 metals (Al, As, Cd, Co, Cu, Fe, Mn, Mo, Se, Tl, and Zn) in blood. A positive association was identified between the PC represented by Al, Cd, and Mn and risk of first ischemic stroke, whereas an inverse association was identified for the PC with high loadings for Fe and Se. The study by Xiao et al. [66] included a total of 1035 cases and 1035 controls for ischemic stroke and 269 cases and 269 controls for hemorrhagic stroke in the Dongfeng-Tongji cohort in China. Out of 18 metals measured in blood (Al, As, Ba, Co, Cu, Pb, Mn, Hg, Mo, Ni, Rb, Se, Sr, Tl, Ti, W, V, and Zn), elastic net selected Cu, Mo, and Se as metals associated with ischemic stroke and Rb and Se as metals associated with hemorrhagic stroke. The regression coefficients for these selected metals from the elastic net model were used as weights and summed to construct predictive plasma metal scores. In adjusted conditional logistic regression model, the calculated predictive plasma metal scores were positively associated with risk of ischemic stroke (OR: 1.37; 95% CI: 1.20, 1.56) and hemorrhagic stroke (OR: 1.53; 95% CI: 1.16–2.01).

Other cardiovascular outcomes evaluated included carotid intima-media thickness, AD, arterial stiffness of peripheral arteries, and global metabolic risk score. Liberda et al. [54] examined 43 contaminants, including 10 metals (As, Pb, Cd, Hg, Se, Co, Cu, Mo, Ni, and Zn) measured in blood, in relation to carotid intima-media thickness in the Environment-and-Health Study in the Eeyou Istchee territory of Quebec, Canada. Using PCA as their multipollutant approach, Of PCs identified, only PC-5, which had a high loading for Ni, was statistically significantly associated with increased carotid intima-media thickness. Liu et al. [68] investigated metal mixture associations with risk of AD, employing WQSR and BKMR as their multipollutant approaches. The study population included a total of 310 adults in Nanjing, China, and concentrations of 10 metals (Mo, Tl, Cu, Cs, Ba, Pb, Cr, Mn, Co, and Ni) were measured in blood. The WQSR analysis indicated an elevated risk of AD for each quantile increase in the metal mixture (coefficient: 3.49, 95% CI: 2.25–5.28) and identified Cu, Ni, and Mo as the main mixture components contributing to this association. Similarly, the BKMR analysis showed a positive association between the metal mixture and risk of AD, pinpointing Cu and Mo as the most influential contributors based on their PIPs. The study by Yang et al. [48] was conducted among 127 adults in Wuhan, China. Out of the 23 metals (Al, Sb, As, Ba, Cd, Cr, Co, Cu, Fe, Pb, Mn, Mo, Ni, Rb, Se, Sr, Tl, Sn, Ti, W, U, V, and Zn) measured in urine, LASSO identified Ti and Co as important predictors of arterial stiffness of peripheral arteries. Interestingly, the inverse association between Ti and a measure of arterial stiffness of peripheral arteries was more pronounced among never-smokers. However, a study of 548 participants from a prospective birth cohort in Mexico by Kupsco et al. [55] did not find evidence for a cumulative association between 11 metals (As, Cd, Co, Cr, Cs, Cu, Mn, Pb, Sb, Se, and Zn) measured in maternal blood during pregnancy and global metabolic risk score among the offspring.

### 3.5. Statistical Methods for Analyzing Effects of Chemical Mixtures

Across studies, the most frequently used multipollutant approaches included PCA, BKMR, and WQSR. Importantly, the goals and assumptions of these modeling techniques differ (Table S3). PCA is an unsupervised, dimension reduction technique which aims to explain as much of the total variance in the data as possible using the minimum number of components [70]. While PCA is widely employed, it has important limitations, including: (1) generating components that may not be easily interpreted, (2) reliance on the researcher’s judgement for deciding the number of components to retain, and (3) the possibility of selecting environmental chemicals that may be irrelevant to the outcomes of interest, as it is an unsupervised approach [71]. In contrast, BKMR is a supervised, semi-parametric method that examines exposure-response relationships using a non-parametric kernel function, while parametrically accounting for covariates. BKMR can evaluate the health impacts of environmental chemicals both individually and jointly, allowing for non-linearity and non-additivity in these associations. BKMR also includes a hierarchical variable selection function, which allows researchers to group chemical mixture components using prior knowledge to determine their group-wise relationships with health outcomes. In addition, PIPs are calculated to estimate the relative importance of each element in the overall effect of the chemical mixture. However, BKMR often requires a large sample size due to its non-parametric property [24,70,72]. WQSR is another supervised mixture analysis method which estimates an empirically weighted index of all pollutants in the mixture. WQSR reduces the impact of outliers by transforming continuous exposures into quantiles. In addition to evaluating joint impacts of multiple pollutants on an outcome, WQSR can be utilized as a variable selection tool, as weights are assigned to each mixture member and reflect the contribution of each mixture component. An important limitation of WQSR is that, unlike BKMR, interactions must be hard-coded into models using prior information. WQSR also assumes directional homogeneity in the mixtures effect (i.e., all mixture components are either positively or negatively associated with the outcome), which may not be a reasonable assumption when both toxic and essential metals are being evaluated. It also assumed linear exposure-response relationships and non-additivity [25,70,73]. The strong assumptions of WQSR make it less flexible than BKMR, but it is also less computationally intensive and can be very useful in situations where those assumptions can be met. Of note, quantile g-computation, a newer approach, relaxes the assumptions of directional homogeneity, linearity, and additive relationships [26]. This method was also shown to be more robust and to perform better than WQSR in a range of simulation studies [26,74].

Despite the multiple existing mixtures analysis techniques, no single method can answer all questions and there is no gold standard for quantifying health effects of environmental chemical mixtures [69,70]. For instance, among the included studies using both BKMR and WQSR, Liu et al. [68] showed that these mixtures analysis methods identified a similar set of metals as the major contributors for risk of AD, whereas Li et al. [53] and Shih et al. [57] observed that these approaches selected very different sets of metals as predictors of lipid markers or BP, respectively. Therefore, well-defined research questions should guide the selection of the best statistical method for analyzing the effect of chemical mixtures. For complex research questions that cannot be addressed by a single mixture analysis technique, more than one method might be employed, maximizing the different strengths of each tool. As this area of methods development continues to rapidly evolve, it is also critical to monitor the availability of new methods [29]. Potential bias amplification due to mutual adjustment for exposure components within a mixture is also an important consideration for environmental mixture studies [75]. Solutions for minimizing co-exposure amplification bias include examining the correlation structure of the exposures prior to the analysis and employing an instrumental variable approach if needed.

### 3.6. Challenges and Opportunities in the Study of Metal Mixtures and CVD Risk Factors and Outcomes

Several opportunities and challenges for future studies should be noted. First, most studies on this topic have been conducted in either the USA or China. To enhance generalizability and explore potentially different dose-response relationships, research should be extended to lower income countries where metal concentrations and exposure sources often differ [76]. Second, while approximately one third of the retrieved studies were conducted in prospective cohort studies, many included studies were cross-sectional in design. These studies may be subject to reverse causation. For example, hypertension increases risk for chronic kidney disease [77], and renal dysfunction in turn may affect the urinary excretion of metals [78]. Therefore, future prospective studies evaluating metal mixture impacts on CVD risk factors and outcomes are strongly recommended. Third, few studies have investigated the impact of metal mixtures on cardiovascular outcomes beyond BP and dyslipidemia. This may be due, in part, to the logistical ease of studies with short duration of follow-up or due to a lack of understanding of the etiologically relevant exposure window for outcomes such as MI or stroke. Regardless, further research is warranted to assess the association between metal mixtures exposure and risk of CVDs less frequently explored, and findings from the current review provides a strong foundation from which to generate new hypotheses. Fourth, to account for potential intra-individual variation in metal concentrations and identify windows of susceptibility, repeated measures of multiple metal exposures, which has rarely been considered, is important and recommended for future studies [4]. Fifth, several of the studies in our review included non-metal exposures in their mixtures [38,43,51,54]. While some of these studies observed significant associations between metal exposures and CVD risk factors and outcomes after accounting for other chemical co-exposures [43,51,54], an exposome study which evaluated 89 different chemicals did not find evidence for an association between metal mixtures and childhood BP [38]. Given these findings, and the potentially complicated interactions between metal and non-metal exposures, future studies may also consider other relevant chemical or non-chemical exposures within their mixtures. New analysis methods which are both reproducible and efficient are also greatly needed to accommodate the growing body of biomedical data, including high-dimensional exposome data [23].

Importantly, many of the studies included in this review may have been impacted by residual confounding. Age, sex, and smoking status are key covariates that need to be considered when evaluating metal mixture impacts on risk for CVDs [17]. While most of the included studies adjusted for age and sex, many studies did not account for smoking status. Fish consumption is also an important potential confounder, as certain metals and metal species such as Hg and arsenobetaine are abundant in fish and seafood [79], and fish also contain essential nutrients that may reduce the risk of CVDs [80,81]. Sensitivity analyses restricting to non-fish consumers are therefore important for studies assessing the cardiotoxicity of metal mixtures, especially when arsenic and mercury are being evaluated [82]. Furthermore, while total As concentrations in urine and blood are frequently used as biomarkers of As exposure [83], this measure may largely reflect organic arsenicals in populations which consume fish and seafood intake, rather than inorganic arsenic [84,85]. Speciation of arsenic and/or the utilization of biospecimens which primarily reflect inorganic arsenic, such as toenail clippings, is therefore critical for studies evaluating arsenic as a mixture component [86]. Careful biospecimen selection is also essential for studies examining Hg exposure within their mixtures, as Hg concentrations in blood, hair, and nails predominantly reflect methyl Hg from fish and seafood consumption [87,88,89,90], whereas urinary Hg primarily reflects inorganic Hg [91,92].

A few of the included studies conducted stratified analysis by potential effect modifiers, including smoking status [48], race [47], and child sex [57]. Identification of effect modification in epidemiological studies can lead to the discovery of biological mechanisms underlying the associations of interest and the development of more targeted interventions [93]. Some of the included studies also reported potential interactions among the metals within the mixture, such as Cd-Zn [53,59] or between toxic and essential metal groups [61]. Knowledge of potential metal interactive effects can be beneficial for developing future interventions. For example, Zn—an essential metal—can alleviate Cd’s pathogenic impacts [94,95]. Recent advancements in mixture analysis methods have enabled estimating and testing interactions among exposures in their effects on outcomes [23].

Finally, to the best of our knowledge, there is currently no meta-analysis technique which can combine findings from multipollutant studies. However, given the growing literature in this field, the development of meta-analysis methods for studies investigating the health impacts of chemical mixtures should be a future research priority.

## 4. Conclusions

To our knowledge, this is the first scoping review of the literature investigating metal mixture impacts on CVD risk factors and outcomes. CVDs are the leading cause of morbidity and mortality worldwide and are anticipated to remain so in the coming decades [96]. Given the potential cardiotoxicity of metal exposures, understanding the role of metal mixture exposures in the development of CVDs is of public health importance. Recognizing the importance of studying complex mixtures of environmental chemicals and their effects on human health, the NIEHS has developed the Combined Exposure and Mixtures Working Group [97] and the PRIME program [23,31]. This increased focus and dedication of resources has helped the field of environmental epidemiology unravel the more complete mechanisms underlying the cardiotoxicity of metal mixtures [97]. The collective evidence currently suggests possible cardiotoxic effects of multiple metals, with many studies identifying important differences between single-pollutant and multipollutant models. However, given that meta-analysis methods for multipollutant studies do not currently exist, and considering important limitations of the original studies, such as a lack of repeated measures of metal mixtures, our conclusions need to be interpreted with caution. Although studying the cardiotoxicity of metal mixture exposures is challenging, it is critical as most individuals are exposed simultaneously to multiple metals; studies utilizing multipollutant models can therefore help policy makers set up more appropriate, evidence-based CVD prevention strategies and standards to reduce metal exposures. There is therefore a need for additional high-quality prospective studies investigating the impacts of metal mixture exposures on CVD risks in populations exposed to a wider range of metal exposures, as well as studies with repeated measures of multiple metal exposures to identify key windows of susceptibility.

## Figures and Tables

**Table 1:** Populations, Exposures, Comparators, and Outcomes (PECO) statement.

Populations	Exposures	Comparators	Outcomes
Healthy humans without restrictions based on age, sex, race, or country. Studies which focused on ill patients and occupational studies were excluded	Exposure to metals, including both toxic and essential elements. To be included, a minimum of three metals needed to have been considered and exposures must have been measured at the individual level in human biological samples (e.g., serum, urine, or toenails)	The comparators differed across studies depending on the mixture analysis methods used	Cardiovascular risk factors or outcomes, including stroke, myocardial infarction, coronary heart disease, hypertension, dyslipidemia, or pregnancy hypertension. Outcomes could be either self-reported, extracted from medical records, or based on investigator collected measurements

**Table 2:** Studies included in the review.

Source	Study Location ^a^	Study Design	Study Population ^b^	Metals Included	Exposure Matrix	Outcome(s) Studied	Mixture Analysis Method(s)	Covariates	Summary of Main Findings
*BP and hypertension (n = 15)*									
Park et al. 2017	USA	CS	9664 adults (4911 females/4753 males)	Pb, Cd, Hg, Sb, As, Ba, Co, Cs, Mo, Tl, W, and U	Blood and urine	SBP, DBP, and hypertension	ERS (AENET-I)	Age, sex, race/ethnicity, education, smoking status, and BMI	ERS based on AENET-I performed better than other approaches and included Cd, Co, and Ba. ERS showed significant associations with SBP, DBP, and hypertension
Wang et al. 2018	USA	CS	9537 adults (4841 females/4696 males)	Cd, Pb, Hg, Sb, As, Ba, Co, Cs, Mo, Tl, W, and U	Blood and urine	Hypertension	ERS (Adaptive Elastic Net)	Age, sex, race/ethnicity, education, smoking status, physical activity, and NHANES cycles	ERS included seven main effects (blood Pb, Cd, and Hg, and urinary monomethylarsonic acid, Ba, Hg, and Tl), four squared terms, and seven pairwise interactions The association between ERS and hypertension was significant (p < 0.05)
Kupsco et al. 2019	Mexico City, Mexico	CO	548 mother-child pairs (272 females/276 males)	As, Cd, Co, Cr, Cs, Cu, Mn, Pb, Sb, Se, and Zn	Blood	SBP and DBP	BKMR	Maternal age, education, socioeconomic status, parity, environmental tobacco smoke, and date of follow-up visit (for HbA1c, global risk score, non-HDL cholesterol, SBP and DBP outcomes only). Birth weight, gestational age, sex, and pre-pregnancy BMI included as covariates in sensitivity analyses	No effect of the metal mixture on BP was observed
Warembourg et al. 2019	Europe	CO	1277 mother-child pairs (580 females/697 males)	As, Cd, Co, Cs, Cu, Hg, Mn, Mo, Pb, and Tl (out of 89 prenatal exposures)	Blood	SBP and DBP	DSA	Cohort, maternal age, maternal education level, maternal pre-pregnancy BMI, parity, parental country of birth, child age, child sex, and child height	The DSA method selected 5 and 2 prenatal exposures for SBP and DBP in childhood, respectively. None of these exposures included metals
Castiello et al. 2020	Granada, Spain	CS	133 male adolescents	As, Cd, Hg, Ni, Pb, Mn, and Cr	Urine	SBP, DBP, elevated BP, and PP	PCA	Age, serum TG, HDL, LDL, and BMI	PC-1 included Ni, Cr, and Mn; PC-2 included As and Hg; and PC-3 included Cd and Pb A suggestive association found between PC-2 and increased SBP
Desai et al. 2021	USA	CS	1642 child or adolescents (824 females/818 males)	Pb, Hg, As, and Cd	Blood and urine	SBP, DBP and PP	BKMR	Age, sex, race, BMI, total energy intake, NHANES cycle, education of household head, and income to poverty ratio	A suggestive inverse association of the mixture of low-level Pb, Hg, As, and Cd with DBP was found, but not with the other outcomes No individual association detected No indication of effect modification observed
Everson et al. 2021 *	USA	CS	2413 adults (female to male ratio not provided)	Ba, Cd, Co, Cs, Mo, Sb, Tl, W, and Pb	Blood and urine	SBP and DBP	Regression tree	Age and its squared term, race, sex, BMI, and smoking status	Having the higher concentrations of Sb, Cd, W, and Pb was associated with higher predicted SBP Having the higher concentrations of Cs and Mo was associated with lower SBP and DBP High Sb was particularly predictive of BP among non-Hispanic white adults, whereas Cd was particularly relevant of BP among non-Hispanic black adults
Howe et al. 2021	Heraklion, Greece	CO	176 mother-child pairs (78 females/98 males)	Mg, Co, Se, Mo, As, Cd, Sb, and Pb	Urine	SBP, DBP, BP change, and elevated BP	BKMR	Maternal age, maternal education, maternal pre-pregnancy BMI, maternal smoking during pregnancy, child’s sex, child’s age, and child’s height	Mo and Co were associated with increased SBP and DBP at baseline (age 4). J-shaped associations were identified Cd was inversely associated with DBP at baseline (age 4) Co was associated with lower per-year increases in both SBP and DBP from ages 4 to 11 Mo was associated with lower per-year increases in DBP from ages 4 to 11 Mg was associated with higher per-year increases in both SBP and DBP from ages 4 to 11, but not with BP at baseline (age 4) Mo and Pb were associated with BP at age 11 (J-shaped) A possible synergistic interaction between Mo and Pb was shown for BP at ages 4 and 11
Kim and Park. 2021	South Korea	CS	10,566 adults (5843 females/4723 males)	Pb, Hg, and Cd	Blood	SBP, DBP, and hypertension	WQSR	Age and sex	A quartile increase in the WQSR index was significantly associated with hypertension risk [OR = 1.29 (95% CI: 1.19–1.40)] Pb contributed most to the WQSR index (weight: 0.662 compared with 0.232 for Hg and 0.106 for Cd)
Shih et al. 2021	Bangladesh	CO	491 mother-child pairs (242 females/249 males)	Al, As, Cd, Cr, Co, Cu, Fe, Pb, Mn, Hg, Mo, Ni, Se, Sn, U, V, and Zn	Toenail samples	BP	PCA, WQSR, and BKMR	Maternal age, maternal education, passive tobacco smoke exposure during pregnancy, child age, child sex, and height	Among boys, PC-2 was heavily loaded for Cu, Zn, Se, Cd, and Pb, and PC-3 was primarily loaded for Hg; among girls, PC-3 was characterized by Sn, Se, and Zn Among boys, PC-2 was associated with higher DBP, whereas PC-3 was associated with lower DBP; among girls, PC-3 was associated with lower SBP The WQSR positive index and DBP were associated in the whole study population and among boys. Among the whole study population, Se, Ni, and Zn were main contributors to the positive WQSR index; among boys, Mo, Se, Ni, Cd, Pb, and As were meaningful contributors Using BKMR, Sn was the most important metal in the mixture in relation to SBP, whereas Hg, Se, and Mo were important with DBP in the whole population; among boys, Se, Hg, Mo, and Cr contributed most to DBP; among girls, Sn and Cu in association with SBP and DBP, respectively. Linearity assumption was generally met, except for the associations of Sn with SBP, and Cu and Se with DBP in girls No evidence of pairwise interactions observed
Xu et al. 2021	USA	CS	957 adults (246 females/711 males)	Cd, Pb, Hg, Mn, and Se	Blood	BP and hypertension	Quantile g-computation	Age, sex, race, educational attainment, and household income level	A quartile increase in the mixture was not significantly associated with the prevalence of hypertension (OR: 0.96; 95% CI: 0.73–1.27) Mn and Se were positively weighted (summary OR:1.15), whereas Pb, Hg, and Cd were negatively weighted (summary OR: 0.84) within the mixture in relation to risk of hypertension Mn (0.62) and Pb (0.45) had the greatest proportional positive or negative contribution to the joint effect, respectively
Yao et al. 2021	USA	CS	9662 adults (4910 females/4752 males)	As, Pb, Cd, and Hg	Blood and urine	SBP, DBP, and hypertension	K-medoids	Age, gender, ethnicity, education, smoking status, and BMI	The k-medoids algorithm categorized the study population into 2 groups according to either blood or urinary levels of heavy metals The “high-exposure” group based on blood levels was significantly associated with SBP
Zhang et al. 2021	Boston, USA	CO	1194 mother-child pairs (603 females/591 males)	Pb, Hg, Cd, Se, and Mn	Blood	SBP and DBP	BKMR	Maternal age, at delivery, race/ethnicity, educational level, pre-pregnancy body mass index, and cigarette smoking history	Joint association not found The hierarchical variable selection indicated that trace elements were more strongly associated with SBP than heavy metals. Among the heavy metals, Pb had the largest conditional PIP; among the trace elements, Se had the larger conditional PIP than Mn In the BKMR individual analysis, Se and Mn were inversely associated with child SBP, but no association detected for DBP
Zhong et al. 2021 *	Tongling, Maanshan, and Chizhou, China	CO	1303 adults (726 females/577 males)	As, B, Ba, Bi, Cd, Co, Cr, Cu, Fe, Li, Mg, Mn, Mo, Rb, Se, Sr, and Zn	Urine	Hypertension	BKMR (Cd, Cu, Mg, Mo, and Zn included)	Age, sex, smoking, drinking, BMI and BP at baseline	Significant joint effect of the five metals on hypertension observed (at or above their 55th percentile compared with their median values) Three groupPIPs > 0.5. The condPIPs of Mo (0.67) and Zn (0.91) were the highest in their groups, respectively Cd associated with increased odds of hypertension, holding other metals constant at their medians Potential interaction between Cd and Zn was observed for elevated risk of hypertension
Zuk et al. 2021	Quebec, Canada	CS	759 adults (447 females/312 males)	Cd, Hg, Pb, and Se (along with other POPs)	Blood	BP and hypertension	PCA	Age, sex, total lipids, smoking status, and BMI	PC-1 was highly and positively loaded for all PCBs, OCs, and moderately loaded on Hg. PC-2 was moderately loaded on Pb PC-1 was associated with stage 2 hypertension
*Preeclampsia (n = 3)*									
Bommarito et al. 2019 *	Boston, USA	CO	28/355 pregnant women	As, Ba, Cd, Cu, Hg, Mn, Mo, Ni, Pb, Se, Sn, Tl, Zn, Be, Cr, U, and W	Urine	Preeclampsia	PCA	Smoking during pregnancy, race, educational attainment, insurance status, infant sex, ART, calcium supplementation, pre-pregnancy BMI, and gestational age at study visit	PC-1 was characterized by essential metals (Cu, Se, and Zn); PC-2 characterized by toxic metals (Cd, Mn, and Pb); and PC-3 characterized by seafood-associated metals No main associations observed between the three PCs and preeclampsia The association between PC-2 and preeclampsia was significant among individuals with low levels of PC-1
Wang et al. 2020	Taiyuan, China	CC	427/427 pregnant women	Cr, Co, Ni, As, Cd, Sb, Hg, and Pb	Blood	Preeclampsia	WQSR and PCA	Matched by age, residence area, and conception time, and adjusted for education, household monthly income per capita, gestational age, and pre-pregnancy BMI	Individuals with PC-2 scores in the highest tertile (high loadings for Cr and As) had increased prevalence of preeclampsia compared with those in the lowest tertile (OR: 1.59; 95% CI: 1.10–2.31) Individuals with PC-3 scores in the highest and middle tertiles (high loadings for Pb and Hg) had increased prevalence of early onset preeclampsia compared with those in the lowest tertile (OR: 2.19; 95% CI: 1.34–3.60 and OR: 2.48; 95% CI: 1.45–4.25 for the middle or highest tertile vs. lowest tertile, respectively) In the WQSR analysis, OR for tertile sum increase in metals and preeclampsia: 1.68 (95% CI: 1.20–2.33); largest weights for Cr (0.447), Hg (0.216), Pb (0.183), and As (0.139)
Liu et al. 2021	USA (12 clinical sites across the nation)	CO	1832 women in the longitudinal analysis, 1688 women in the cross-sectional analysis	Ba, Cs, Sb, Co, Cu, Mo, Se, and Zn	Blood	Baseline SBP, DBP, and rates of weekly BP changes over pregnancy	BKMR	Maternal age at enrollment, maternal race/ethnicity, maternal educational achievement, marital status, parity, self-reported pre-pregnancy BMI, pre-pregnancy to 1st trimester moderate to vigorous level of physical activity, and gestational age at chemical measurement	Holding all chemicals at their 90th percentile was associated with a 1.61 mmHg (95% CI: 0.41, 2.81) higher SBP and a 1.09 mmHg (95% CI: 0.10, 2.09) higher DBP at baseline compared to holding all chemicals at their median levels Accounting for other chemicals within the mixture, each IQR increment in Cu was associated with a 0.67 mmHg (95% CI: 0.02, 1.32) higher SBP and a 0.60 mmHg (95% CI: 0.08, 1.12) higher DBP at baseline; each IQR increment in Se was associated with a 0.67 mmHg (95% CI: 0.05, 1.29) higher SBP but not DBP No interactions observed
*Dyslipidemia and serum lipid levels (n = 5)*									
Park et al. 2014	USA	CS	Stage 1: 10,818 adults (5789 females/5029 males) Stage 2: 4615 adults (2395 females/2220 males)	Pb, Cd, Hg, and As out of 149 pollutants	Blood and urine	TC, HDL, LDL, and TG	ERS (EWAS)	Age, gender, race/ethnicity, education, BMI, and serum micronutrients	The EWAS identified 13 pollutants associated with total cholesterol, 9 for HDL, 5 for LDL and 27 for triglycerides In the multi-pollutant analysis, blood Pb and Cd were associated with increased total cholesterol Blood Pb was also associated with greater HDL and LDL Blood Cd was associated with greater triglycerides. Urinary Cd was associated with lower HDL and higher LDL Hg in blood or urine and urinary arsenobetaine were associated with decreased triglycerides Urinary Sb was associated with lower HDL
Kupsco et al. 2019	Mexico City, Mexico	CO	548 mother-child pairs (272 females/276 males)	As, Cd, Co, Cr, Cs, Cu, Mn, Pb, Sb, Se, and Zn	Blood	Non-HDL cholesterol, TG, leptin, adiponectin	BKMR	Maternal age, education, socioeconomic status, parity, environmental tobacco smoke, and date of follow-up visit (for HbA1c, global risk score, non-HDL cholesterol, SBP and DBP outcomes only). Birth weight, gestational age, sex, and pre-pregnancy BMI included as covariates in sensitivity analyses	Higher Se was associated with lower TG Sb and As were associated with lower leptin No interaction among the metals or non-linear responses detected
Zhu et al. 2021	West Anhui, China	CS	1013 adults (552 females/461 males)	Sr, Cd, Pb, V, Al, Co, and Mn	Blood	Dyslipidemia	PCA	Gender, age, education level, per capita income, BMI, occupation, smoking, drinking, exercise, and disease history of hypertension, diabetes, stroke, and coronary heart disease	PC-1 was represented by V, Al, and Co; PC-2 (Sr, Cd, and Pb); and PC-3 (Mn) PC-2 was positively associated with the prevalence of dyslipidemia, whereas PC-1 was inversely associated No association found between Mn (PC-3) and dyslipidemia
Jiang et al. 2021	Hubei, China	CO	2947 adults (1473 females/1474 males)	Al, Sb, As, Ba, Co, Cu, Pb, Mn, Mo, Ni, Rb, Se, Sr, Tl, Ti, V, and Zn	Blood	Incident dyslipidemia	PCA	Age, gender, BMI, education level, smoking status, drinking status, physical activity, fasting blood glucose, eGFR, hypertension, family history of dyslipidemia, and measurement batch	PC-1 characterized by combined exposure to Al, As, Ba, Pb, V, and Zn; PC-2 (Sb, Co, and Tl); PC-3 (Cu, Rb, and Se); PC-4 (Ti and V); and PC-5 (Ni) Compared with the first quartile of the scores of PC-1, the fourth quartile of PC-1 was associated with elevated risk of dyslipidemia (aOR: 1.40; 95% CI: 1.07, 1.84). PC-1 also associated with higher risks of low HDL-C and high LDL-C PC-3 was associated with the higher TC risk and lower HDL-C risk.
Li et al. 2021 *	Hunan, China	CS	564 (293 females/271 males) and 637 adults (449 females/188 males) from Shimen and Huayuan, respectively	Al, As, Ba, Cd, Co, Cr, Cu, Fe, Mn, Mo, Ni, Rb, Sb, Se, Sn, Sr, Ti, Tl, U, W, V, and Zn	Blood and urine	The concentrations of TG, TC, HDL-C, and LDL-C	WQSR and BKMR	Age, gender, BMI, smoke, drink, physical activity, education, ethnicity, income level, hypertension, family history of hyperlipidemia, and eGFR	WQSR index associated with lipids, except for HDL-C, for both positively and negatively constrained models. In the grouped WQSR analysis, essential metals were positively associated with all lipid markers, except for HDL-C in Huayuan area. Toxic metals were negatively associated with lipids, except for HDL-C and LDL-C in Huayuan area. BKMR suggested no joint effects Potential non-linear relationship was observed between the metal mixture and TC and LDL-C levels The associations of exposure to Zn or Ti with TG levels were consistently found in both areas, but the other associations varied by sites An inverse U-shaped association of Fe with LDL-C levels were detected in Huayuan area Possible interaction between Zn and Cd was detected in association with LDL-C in Huayuan area
*CVD outcomes (n = 8)*									
Domingo-Relloso et al. 2019	Valladolid, Spain	CO	1171 adults (566 females/605 males	Sb, Ba, Cd, Cr, Co, Cu, Mo, V, and Zn	Urine	Combined endpoint for incident coronary heart disease and stroke	BKMR-P	Sex, education, smoking status, cumulative smoking dose, urine cotinine, age, estimated GFR, residence, HDL cholesterol level, total cholesterol level, dyslipidemia treatment, hypertension treatment, diabetes mellitus of type 2 and systolic pressure	Cu, Zn, Sb, Cd, Sr, and V were associated with cardiovascular incidence, with Cd and Sb showing the highest PIPs
Kupsco et al. 2019	Mexico City, Mexico	CO	548 mother-child pairs (272 females/276 males)	As, Cd, Co, Cr, Cs, Cu, Mn, Pb, Sb, Se, and Zn	Blood	Cardio-metabolic component scores	BKMR	Maternal age, education, socioeconomic status, parity, environmental tobacco smoke, and date of follow-up visit (for HbA1c, global risk score, non-HDL cholesterol, SBP and DBP outcomes only). Birth weight, gestational age, sex, and pre-pregnancy BMI included as covariates in sensitivity analyses	No effect of the metal mixture on global cardio-metabolic risk score was observed
Liberda et al. 2019	Quebec, Canada	CS	535 adults (299 females/236 males)	As, Pb, Cd, Hg, Se, Co, Cu, Mo, Ni, and Zn out of 43 contaminants	Blood	Carotid intima-media thickness	PCA	Age, sex, smoking status, BMI, SBP, LDL, Apo-B, triglycerides, TNF-α, hs-CRP, and ox-LDL	Metals were mainly loaded on PC-4 and PC-5, including Ni, Se, and Cd Carotid intima-media thickness was significantly associated with PC-5, which was mostly represented by Ni
Wen et al. 2019	Shenzhen, China	CC	1277/1277 adults (548 females/729 males for both controls and cases)	Al, As, Cd, Co, Cu, Fe, Mn, Mo, Se, Tl, and Zn	Blood	First ischemic stroke	PCA	Matched by age and sex, with adjustment for BMI, smoking, alcohol drinking, hypertension, diabetes, and hyperlipidemia	The first PC, represented by Al, Cd, and Mn, was positively associated with risk of ischemic stroke The second PC, represented by Fe and Se, was inversely associated with risk of ischemic stroke
Xiao et al. 2019	Dongfeng, China	CC	1035/1035 adults (382 females/653 males for both controls and cases) for ischemic stroke; 269/269 adults (112 females/157 males) for hemorrhagic stroke	Al, As, Ba, Co, Cu, Pb, Mn, Hg, Mo, Ni, Rb, Se, Sr, Tl, Ti, W, V, and Zn	Blood	Incident stroke	Elastic net regression	Matched on age, sex, and blood sampling date, and adjusted for BMI, smoking, drinking staus, regular exercise, family history of stroke, hyperlipidemia, diabetes mellitus, and hypertension	Elastic net selected Cu, Mo, and Se for ischemic stroke, and Rb and Se for hemorrhagic stroke. The selected metals were included in predictive plasma metal scores Per one IQR increase in predictive plasma metal scores, the adjusted OR was 1.37 (95% CI: 1.20, 1.56) for ischemic stroke and 1.53 (95% CI: 1.16, 2.01) for hemorrhagic stroke
Cabral et al. 2021	Potsdam, Germany	CCO	2087 adults (1304 females/783 males)	Mn, Fe, Cu, Zn, I, and Se	Blood	CVD outcomes (incident MI and stroke)	PCA	Age, sex, education, BMI, waist circumference, smoking status, overall leisure-time physical activity, alcohol consumption, prevalent hypertension, anti-hypertensive and lipid-lowering medication, vitamin and mineral preparations, and dietary quality	PC-1 was mainly related to higher concentrations of Mn, Fe, and Zn, whereas PC-2 to Cu, I, and Se Only PC-2 was associated with risk of developing CVD
Liu et al. 2021	Nanjing, China	CC	127/183 adults (30 females/97 males for cases; 46 females/137 males for controls)	Mo, Tl, Cu, Cs, Ba, Pb, Cr, Mn, Co, and Ni	Blood	AD	BKMR and WQSR	Matched by age and sex, with adjustments for BMI, education level, smoking status, drinking status, BP, history of hypertension, subtype of AD, and the WBC count	The WQSR analysis suggested elevated risk of AD per every unit increase in the metal mixture index (coefficient = 3.49, 95% CI: 2.25, 5.28), with Cu, Ni, and Mo as the main contributors The BKMR analysis indicated a significantly positive trend for the association of the metal mixture with AD, with Cu and Mo showing the greatest PIPs
Yang et al. 2021 *	Wuhan, China	Panel study	127 adults (90 females/37 males)	Al, Sb, As, Ba, Cd, Cr, Co, Cu, Fe, Pb, Mn, Mo, Ni, Rb, Se, Sr, Tl, Sn, Ti, W, U, V, and Zn	Urine	Arterial stiffness of peripheral arteries	LASSO	Age, sex, BMI, smoking status, drinking status, education, physical activity, hypertension, hyperlipidemia, diabetes, heart rate, and community	Ti and Co were identified as the important predictors of ABI In a stratified analysis, the inverse associations between urinary metals and ABI was more pronounced among never-smokers

ABI, ankle-brachial index; AD, aortic dissection; ART, assisted reproductive technology; BMI, body mass index; BP, blood pressure; CRP, C-reactive protein; CVD, cardiovascular disease; DBP, diastolic blood pressure; eGFR, estimated glomerular filtration rate; GGT, gamma-glutamyl transferase; HbA1c, hemoglobin A1c; HDL, high-density lipoprotein; LDL, low-density lipoprotein; MI, myocardial infarction; NHANES, National Health and Nutrition Examination Survey; OCs, organic compound concentrations; OR, odds ratio; PC, principal component; PCB, polychlorinated biphenyls; PIP, posterior inclusion probability; POP, persistent organic pollutant; PP, pulse pressure; SBP, systolic blood pressure; TC, total cholesterol; TG, triglyceride; TNF, tumor necrosis factor; WBC, white blood cells. * Studies that detected potential interactions within a mixture. ^a^ We had to use broader terms, such as “USA” and “Europe”, for some studies because these were conducted across all the USA states or European countries. ^b^ For the case-control studies, number of cases/number of controls.

## Data Availability

Not applicable.

## Supplementary Materials

The following supporting information can be downloaded at: https://www.mdpi.com/article/10.3390/toxics10030116/s1, Table S1: Search strategy; Table S2: Metal concentrations from the studies included in this review; Table S3: Comparisons of the mixtures analysis methods most commonly used in this review.

## Abbreviations

List of metal components within the mixtures included in this study.

Al	Aluminum
As	Arsenic
B	Boron
Ba	Barium
Be	Beryllium
Bi	Bismuth
Cd	Cadmium
Co	Cobalt
Cr	Chromium
CS	Cesium
Cu	Copper
Fe	Iron
Hg	Mercury
I	Iodine
Li	Lithium
Mg	Magnesium
Mn	Manganese
Mo	Molybdenum
Ni	Nickel
Pb	Lead
Rb	Rubidium
Sb	Antimony
Se	Selenium
Sn	Tin
Sr	Strontium
Ti	Titanium
Tl	Thallium
U	Uranium
V	Vanadium
W	Tungsten
Zn	Zinc

List of key abbreviations used in Table 2.

Study designs	
CC	Case-control study;
CCO	Case-cohort study;
CO	Prospective cohort study;
CS	Cross-sectional study
Mixture analysis methods	
AENET-I	Adaptive elastic-net with main effects and pairwise interactions
BKMR	Bayesian kernel machine regression
BKMR-P	Probit extension of Bayesian kernel machine regression
DSA	Deletion-substitution-addition algorithm
ERS	Environmental risk score
EWAS	Environment-wide association Study
LASSO	Least absolute shrinkage and selection operator
PCA	Principal component analysis
WQSR	Weighted quantile sum regression
