# Supplementary material for: Exposure to Metal Mixtures in Association with Cardiovascular Risk Factors and Outcomes: A Scoping Review
Source: Toxics. 2022 Mar 1;10(3):116. doi: 10.3390/toxics10030116 (PMC8955637; doi:10.3390/toxics10030116)
Supplement: Supplementary file 1 [file toxics-10-00116-s001.zip › toxics-1577756-supplementary.pdf]

toxics

Supplementary Materials: Exposure to Metal Mixtures in Association with Cardiovascular Risk Factors and Outcomes: A Scoping Review

Gyeyoon Yim, Yuting Wang, Caitlin G. Howe and Megan E. Romano

Table S1. Search strategy

(a) Database: MEDLINE (PubMed)
Publication timeline range: 1998 to October 1, 2021

#	Searches	Results
1	"metals"[tiab] OR "Actinium"[tiab] OR "Aluminum"[tiab] OR "Americium"[tiab] OR "Antimony"[tiab] OR "Arsenic"[tiab] OR "Barium"[tiab] OR "Berkelium"[tiab] OR "Beryllium"[tiab] OR "Bismuth"[tiab] OR "Boron"[tiab] OR "Cadmium"[tiab] OR "Calcium"[tiab] OR "Californium"[tiab] OR "Cesium"[tiab] OR "Chromium"[tiab] OR "Cobalt"[tiab] OR "Copper"[tiab] OR "Curium"[tiab] OR "Einsteinium"[tiab] OR "Fermium"[tiab] OR "Francium"[tiab] OR "Gallium"[tiab] OR "Germanium"[tiab] OR "Gold"[tiab] OR "Hafnium"[tiab] OR "Indium"[tiab] OR "Iridium"[tiab] OR "Iron"[tiab] OR "Lawrencium"[tiab] OR "Lead"[tiab] OR "Lithium"[tiab] OR "Lutetium"[tiab] OR "Magnesium"[tiab] OR "Manganese"[tiab] OR "Mendelevium"[tiab] OR "Mercury"[tiab] OR "Molybdenum"[tiab] OR "Neptunium"[tiab] OR "Nickel"[tiab] OR "Niobium"[tiab] OR "Nobelium"[tiab] OR "Osmium"[tiab] OR "Palladium"[tiab] OR "Platinum"[tiab] OR "Plutonium"[tiab] OR "Polonium"[tiab] OR "Potassium"[tiab] OR "Protactinium"[tiab] OR "Radium"[tiab] OR "Rhenium"[tiab] OR "Rhodium"[tiab] OR "Rubidium"[tiab] OR "Ruthenium"[tiab] OR "Scandium"[tiab] OR "Silicon"[tiab] OR "Silver"[tiab] OR "Sodium"[tiab] OR "Strontium"[tiab] OR "Tantalum"[tiab] OR "Technetium"[tiab] OR "Tellurium"[tiab] OR "Thallium"[tiab] OR "Thorium"[tiab] OR "Tin"[tiab] OR "Titanium"[tiab] OR "Tungsten"[tiab] OR "Uranium"[tiab] OR "Vanadium"[tiab] OR "Yttrium"[tiab] OR "Zinc"[tiab] OR "Zirconium"[tiab] OR "Trace Elements"[Mesh] Filters: from 1998 - 2022	1,988,795
2	"Cardiometabolic Risk Factors"[Mesh] OR "Heart Diseases"[Mesh] OR "Vascular Diseases"[Mesh] OR "Blood Pressure"[Mesh] OR "Lipoproteins, HDL"[Mesh] OR "Lipoproteins, LDL"[Mesh] OR "Pregnancy Complications, Cardiovascular"[Mesh] OR "Hypertension, Pregnancy-Induced"[Mesh] OR "Cardiovascular disease*"[tiab] OR "myocardial infarction*"[tiab] OR "myocardial ischemia*"[tiab] OR "coronary artery*"[tiab] OR “coronary heart”[tiab] OR "cerebrovascular dis*"[tiab] OR "stroke*"[tiab] OR "atherosclerosis*"[tiab] OR "carotid artery"[tiab] OR "peripheral arterial dis*"[tiab] OR "hypercholesterolemia*"[tiab] OR "hyperlipidemia*"[tiab] OR "dyslipidemia*"[tiab] OR “serum lipid”[tiab] OR "pre-eclampsia"[tiab] OR "preeclampsia"[tiab] OR "pregnancy hypertens*"[tiab] OR "HELLP"[tiab] Filters: from 1998 - 2022	1,903,479
3	"mixture*"[tiab] OR "multi-pollutant*"[tiab] OR "multiple metals"[tiab] OR "multi-metal*"[tiab] OR "joint effect*"[tiab] OR "joint association*"[tiab] OR "joint exposure*"[tiab] OR "overall effect*"[tiab] OR "overall association*"[tiab] OR "overall exposure*"[tiab] OR "joint and individual"[tiab] OR "co-pollutant*"[tiab] OR "co-exposure*"[tiab] OR "combined metal*"[tiab] OR "mixed metal*"[tiab] OR "principal component analysis"[tiab] OR "exploratory factor analysis"[tiab] OR "Bayesian Kernel Machine Regression"[tiab] OR "Least Absolute Shrinkage and Selection Operator"[tiab] OR "penalized regression*"[tiab] OR "Weighted Quantile Sum"[tiab] OR "quantile-based g-computation"[tiab] OR "quantile g-computation"[tiab] OR "cluster analysis"[tiab] OR "Bayesian"[tiab] OR "classification and regression tree*"[tiab] OR "random forest*"[tiab] OR "shrinkage-based"[tiab] OR	410,767

	"shrinkage method*"[tiab] OR "variable selection regression*"[tiab] OR "structural equation model*"[tiab] OR "multivariate adaptive regression spline*"[tiab] OR "machine learning"[tiab] Filters: from 1998 - 2022	
4	#1 AND #2 AND #3	1673

(b) Database: Scopus

Publication timeline range: 1998 to October 1, 2021

#	Searches	Results
1	TITLE-ABS ("metals" OR "Actinium" OR "Aluminum" OR "Americium" OR "Antimony" OR "Arsenic" OR "Barium" OR "Berkelium" OR "Beryllium" OR "Bismuth" OR "Boron" OR "Cadmium" OR "Calcium" OR "Californium" OR "Cesium" OR "Chromium" OR "Cobalt" OR "Copper" OR "Curium" OR "Einsteinium" OR "Fermium" OR "Francium" OR "Gallium" OR "Germanium" OR "Gold" OR "Hafnium" OR "Indium" OR "Iridium" OR "Iron" OR "Lawrencium" OR "Lead" OR "Lithium" OR "Lutetium" OR "Magnesium" OR "Manganese" OR "Mendelevium" OR "Mercury" OR "Molybdenum" OR "Neptunium" OR "Nickel" OR "Niobium" OR "Nobelium" OR "Osmium" OR "Palladium" OR "Platinum" OR "Plutonium" OR "Polonium" OR "Potassium" OR "Protactinium" OR "Radium" OR "Rhenium" OR "Rhodium" OR "Rubidium" OR "Ruthenium" OR "Scandium" OR "Silicon" OR "Silver" OR "Sodium" OR "Strontium" OR "Tantalum" OR "Technetium" OR "Tellurium" OR "Thallium" OR "Thorium" OR "Tin" OR "Titanium" OR "Tungsten" OR "Uranium" OR "Vanadium" OR "Yttrium" OR "Zinc" OR "Zirconium") AND (LIMIT-TO (PUBYEAR , 2023) OR LIMIT-TO (PUBYEAR , 2022) OR LIMIT-TO (PUBYEAR , 2021) OR LIMIT-TO (PUBYEAR , 2020) OR LIMIT-TO (PUBYEAR , 2019) OR LIMIT-TO (PUBYEAR , 2018) OR LIMIT-TO (PUBYEAR , 2017) OR LIMIT-TO (PUBYEAR , 2016) OR LIMIT-TO (PUBYEAR , 2015) OR LIMIT-TO (PUBYEAR , 2014) OR LIMIT-TO (PUBYEAR , 2013) OR LIMIT-TO (PUBYEAR , 2012) OR LIMIT-TO (PUBYEAR , 2011) OR LIMIT-TO (PUBYEAR , 2010) OR LIMIT-TO (PUBYEAR , 2009) OR LIMIT-TO (PUBYEAR , 2008) OR LIMIT-TO (PUBYEAR , 2007) OR LIMIT-TO (PUBYEAR , 2006) OR LIMIT-TO (PUBYEAR , 2005) OR LIMIT-TO (PUBYEAR , 2004) OR LIMIT-TO (PUBYEAR , 2003) OR LIMIT-TO (PUBYEAR , 2002) OR LIMIT-TO (PUBYEAR , 2001) OR LIMIT-TO (PUBYEAR , 2000) OR LIMIT-TO (PUBYEAR , 1999) OR LIMIT-TO (PUBYEAR , 1998))	7,143,923
2	TITLE-ABS-KEY ("Trace Elements") AND (LIMIT-TO (PUBYEAR , 2022) OR LIMIT-TO (PUBYEAR , 2021) OR LIMIT-TO (PUBYEAR , 2020) OR LIMIT-TO (PUBYEAR , 2019) OR LIMIT-TO (PUBYEAR , 2018) OR LIMIT-TO (PUBYEAR , 2017) OR LIMIT-TO (PUBYEAR , 2016) OR LIMIT-TO (PUBYEAR , 2015) OR LIMIT-TO (PUBYEAR , 2014) OR LIMIT-TO (PUBYEAR , 2013) OR LIMIT-TO (PUBYEAR , 2012) OR LIMIT-TO (PUBYEAR , 2011) OR LIMIT-TO (PUBYEAR , 2010) OR LIMIT-TO	93,976

	(PUBYEAR , 2009) OR LIMIT-TO (PUBYEAR , 2008) OR LIMIT-TO (PUBYEAR , 2007) OR LIMIT-TO (PUBYEAR , 2006) OR LIMIT-TO (PUBYEAR , 2005) OR LIMIT-TO (PUBYEAR , 2004) OR LIMIT-TO (PUBYEAR , 2003) OR LIMIT-TO (PUBYEAR , 2002) OR LIMIT-TO (PUBYEAR , 2001) OR LIMIT-TO (PUBYEAR , 2000) OR LIMIT-TO (PUBYEAR , 1999) OR LIMIT-TO (PUBYEAR , 1998))	
3	TITLE-ABS-KEY ("Cardiometabolic Risk Factors" OR "Heart Diseases" OR "Vascular Diseases" OR "Blood Pressure" "High Density Lipoprotein*" OR "Low Density Lipoprotein*" OR "Cardiovascular Pregnancy Complication*" OR "Pregnancy Induced Hypertension" OR "Gestational Hypertension" OR "Pregnancy Transient Hypertension") AND (LIMIT-TO (PUBYEAR , 2022) OR LIMIT-TO (PUBYEAR , 2021) OR LIMIT-TO (PUBYEAR , 2020) OR LIMIT-TO (PUBYEAR , 2019) OR LIMIT-TO (PUBYEAR , 2018) OR LIMIT-TO (PUBYEAR , 2017) OR LIMIT-TO (PUBYEAR , 2016) OR LIMIT-TO (PUBYEAR , 2015) OR LIMIT-TO (PUBYEAR , 2014) OR LIMIT-TO (PUBYEAR , 2013) OR LIMIT-TO (PUBYEAR , 2012) OR LIMIT-TO (PUBYEAR , 2011) OR LIMIT-TO (PUBYEAR , 2010) OR LIMIT-TO (PUBYEAR , 2009) OR LIMIT-TO (PUBYEAR , 2008) OR LIMIT-TO (PUBYEAR , 2007) OR LIMIT-TO (PUBYEAR , 2006) OR LIMIT-TO (PUBYEAR , 2005) OR LIMIT-TO (PUBYEAR , 2004) OR LIMIT-TO (PUBYEAR , 2003) OR LIMIT-TO (PUBYEAR , 2002) OR LIMIT-TO (PUBYEAR , 2001) OR LIMIT-TO (PUBYEAR , 2000) OR LIMIT-TO (PUBYEAR , 1999) OR LIMIT-TO (PUBYEAR , 1998))	64,327
4	TITLE-ABS ("Cardiovascular disease*" OR "myocardial infarction*" OR "myocardial ischemia*" OR "coronary artery*" OR "coronary heart" OR "cerebrovascular dis*" OR "stroke*" OR "atherosclerosis*" OR "carotid artery" OR "peripheral arterial dis*" OR "hypercholesterolemia*" OR "hyperlipidemia*" OR "dyslipidemia*" OR "serum lipid" OR "pre-eclampsia" OR "preeclampsia" OR "pregnancy hypertens*" OR "HELLP") AND (LIMIT-TO (PUBYEAR , 2022) OR LIMIT-TO (PUBYEAR , 2021) OR LIMIT-TO (PUBYEAR , 2020) OR LIMIT-TO (PUBYEAR , 2019) OR LIMIT-TO (PUBYEAR , 2018) OR LIMIT-TO (PUBYEAR , 2017) OR LIMIT-TO (PUBYEAR , 2016) OR LIMIT-TO (PUBYEAR , 2015) OR LIMIT-TO (PUBYEAR , 2014) OR LIMIT-TO (PUBYEAR , 2013) OR LIMIT-TO (PUBYEAR , 2012) OR LIMIT-TO (PUBYEAR , 2011) OR LIMIT-TO (PUBYEAR , 2010) OR LIMIT-TO (PUBYEAR , 2009) OR LIMIT-TO (PUBYEAR , 2008) OR LIMIT-TO (PUBYEAR , 2007) OR LIMIT-TO (PUBYEAR , 2006) OR LIMIT-TO (PUBYEAR , 2005) OR LIMIT-TO (PUBYEAR , 2004) OR LIMIT-TO (PUBYEAR , 2003) OR LIMIT-TO (PUBYEAR , 2002) OR LIMIT-TO (PUBYEAR , 2001) OR LIMIT-TO (PUBYEAR , 2000) OR LIMIT-TO (PUBYEAR , 1999) OR LIMIT-TO (PUBYEAR , 1998))	974,815
5	TITLE-ABS ("mixture*" OR "multi-pollutant*" OR "multiple metals" OR "multi-metal*" OR "joint effect*" OR "joint association*" OR "joint exposure*" OR "overall effect*" OR "overall association*" OR "overall exposure*" OR "joint and individual" OR "co-pollutant*" OR "co-exposure*" OR "combined metal*" OR "mixed metal*" OR "principal component analysis" OR "exploratory	1,485,981

	factor analysis" OR "Bayesian Kernel Machine Regression" OR "Least Absolute Shrinkage and Selection Operator" OR "penalized regression*" OR "Weighted Quantile Sum" OR "quantile-based g-computation" OR "quantile g-computation" OR "cluster analysis" OR "Bayesian" OR "classification and regression tree*" OR "random forest*" OR "shrinkage-based" OR "shrinkage method*" OR "variable selection regression*" OR "structural equation model*" OR "multivariate adaptive regression spline*" OR "exposure surface smoothing" OR "novel approach and least-angle regression" OR "machine learning") AND (LIMIT-TO (PUBYEAR , 2023) OR LIMIT-TO (PUBYEAR , 2022) OR LIMIT-TO (PUBYEAR , 2021) OR LIMIT-TO (PUBYEAR , 2020) OR LIMIT-TO (PUBYEAR , 2019) OR LIMIT-TO (PUBYEAR , 2018) OR LIMIT-TO (PUBYEAR , 2017) OR LIMIT-TO (PUBYEAR , 2016) OR LIMIT-TO (PUBYEAR , 2015) OR LIMIT-TO (PUBYEAR , 2014) OR LIMIT-TO (PUBYEAR , 2013) OR LIMIT-TO (PUBYEAR , 2012) OR LIMIT-TO (PUBYEAR , 2011) OR LIMIT-TO (PUBYEAR , 2010) OR LIMIT-TO (PUBYEAR , 2009) OR LIMIT-TO (PUBYEAR , 2008) OR LIMIT-TO (PUBYEAR , 2007) OR LIMIT-TO (PUBYEAR , 2006) OR LIMIT-TO (PUBYEAR , 2005) OR LIMIT-TO (PUBYEAR , 2004) OR LIMIT-TO (PUBYEAR , 2003) OR LIMIT-TO (PUBYEAR , 2002) OR LIMIT-TO (PUBYEAR , 2001) OR LIMIT-TO (PUBYEAR , 2000) OR LIMIT-TO (PUBYEAR , 1999) OR LIMIT-TO (PUBYEAR , 1998))	
6	#1 OR #2	7,188,444
7	#3 OR #4	1,002,568
8	#5 AND #6 AND #7	1743

(c) Database: Web of Science

Publication timeline range: 1998 to October 1, 2021

#	Searches	Results
1	"metals" OR "Actinium" OR "Aluminum" OR "Americium" OR "Antimony" OR "Arsenic" OR "Barium" OR "Berkelium" OR "Beryllium" OR "Bismuth" OR "Boron" OR "Cadmium" OR "Calcium" OR "Californium" OR "Cesium" OR "Chromium" OR "Cobalt" OR "Copper" OR "Curium" OR "Einsteinium" OR "Fermium" OR "Francium" OR "Gallium" OR "Germanium" OR "Gold" OR "Hafnium" OR "Indium" OR "Iridium" OR "Iron" OR "Lawrencium" OR "Lead" OR "Lithium" OR "Lutetium" OR "Magnesium" OR "Manganese" OR "Mendelevium" OR "Mercury" OR "Molybdenum" OR "Neptunium" OR "Nickel" OR "Niobium" OR "Nobelium" OR "Osmium" OR "Palladium" OR "Platinum" OR "Plutonium" OR "Polonium" OR "Potassium" OR "Protactinium" OR "Radium" OR "Rhenium" OR "Rhodium" OR "Rubidium" OR "Ruthenium" OR "Scandium" OR "Silicon" OR "Silver" OR "Sodium" OR "Strontium" OR "Tantalum" OR "Technetium" OR "Tellurium" OR "Thallium" OR "Thorium" OR "Tin" OR "Titanium" OR "Tungsten" OR "Uranium" OR "Vanadium" OR "Yttrium" OR "Zinc" OR "Zirconium" (Title) or "metals" OR "Actinium" OR "Aluminum" OR "Americium" OR "Antimony" OR "Arsenic" OR "Barium" OR "Berkelium" OR "Beryllium" OR "Bismuth" OR "Boron" OR	4,845,259

	"Cadmium" OR "Calcium" OR "Californium" OR "Cesium" OR "Chromium" OR "Cobalt" OR "Copper" OR "Curium" OR "Einsteinium" OR "Fermium" OR "Francium" OR "Gallium" OR "Germanium" OR "Gold" OR "Hafnium" OR "Indium" OR "Iridium" OR "Iron" OR "Lawrencium" OR "Lead" OR "Lithium" OR "Lutetium" OR "Magnesium" OR "Manganese" OR "Mendelevium" OR "Mercury" OR "Molybdenum" OR "Neptunium" OR "Nickel" OR "Niobium" OR "Nobelium" OR "Osmium" OR "Palladium" OR "Platinum" OR "Plutonium" OR "Polonium" OR "Potassium" OR "Protactinium" OR "Radium" OR "Rhenium" OR "Rhodium" OR "Rubidium" OR "Ruthenium" OR "Scandium" OR "Silicon" OR "Silver" OR "Sodium" OR "Strontium" OR "Tantalum" OR "Technetium" OR "Tellurium" OR "Thallium" OR "Thorium" OR "Tin" OR "Titanium" OR "Tungsten" OR "Uranium" OR "Vanadium" OR "Yttrium" OR "Zinc" OR "Zirconium" (Abstract) | Timespan: 1998-01-01 to 2021-10-01 (Publication Date)	
2	"Trace Elements" (Title) or "Trace Elements" (Abstract) or "Trace Elements" (Author Keywords) | Timespan: 1998-01-01 to 2021-10-01 (Publication Date)	36,948
3	"Cardiometabolic Risk Factors" OR "Heart Diseases" OR "Vascular Diseases" OR "Blood Pressure" OR "High Density Lipoprotein*" OR "Low Density Lipoprotein*" OR "Cardiovascular Pregnancy Complication*" OR "Pregnancy Induced Hypertension" OR "Gestational Hypertension" OR "Pregnancy Transient Hypertension" (Title) or "Cardiometabolic Risk Factors" OR "Heart Diseases" OR "Vascular Diseases" OR "Blood Pressure" OR "High Density Lipoprotein*" OR "Low Density Lipoprotein*" OR "Cardiovascular Pregnancy Complication*" OR "Pregnancy Induced Hypertension" OR "Gestational Hypertension" OR "Pregnancy Transient Hypertension" (Abstract) or "Cardiometabolic Risk Factors" OR "Heart Diseases" OR "Vascular Diseases" OR "Blood Pressure" OR "High Density Lipoprotein*" OR "Low Density Lipoprotein*" OR "Cardiovascular Pregnancy Complication*" OR "Pregnancy Induced Hypertension" OR "Gestational Hypertension" OR "Pregnancy Transient Hypertension" (Author Keywords) | Timespan: 1998-01-01 to 2021-10-01 (Publication Date)	331,988
4	"Cardiovascular disease*" OR "myocardial infarction*" OR "myocardial ischemia*" OR "coronary artery*" OR "coronary heart" OR "cerebrovascular dis*" OR "stroke*" OR "atherosclerosis*" OR "carotid artery" OR "peripheral arterial dis*" OR "hypercholesterolemia*" OR "hyperlipidemia*" OR "dyslipidemia*" OR "serum lipid" OR "pre-eclampsia" OR "preeclampsia" OR "pregnancy hypertens*" OR "HELLP" (Title) or "Cardiovascular disease*" OR "myocardial infarction*" OR "myocardial ischemia*" OR "coronary artery*" OR "coronary heart" OR "cerebrovascular dis*" OR "stroke*" OR "atherosclerosis*" OR "carotid artery" OR "peripheral arterial dis*" OR "hypercholesterolemia*" OR "hyperlipidemia*" OR "dyslipidemia*" OR "serum lipid" OR "pre-eclampsia" OR "preeclampsia" OR "pregnancy hypertens*" OR "HELLP" (Abstract) | Timespan: 1998-01-01 to 2021-10-01 (Publication Date)	907,580
5	"mixture*" OR "multi-pollutant*" OR "multiple metals" OR "multi-metal*" OR "joint effect*" OR "joint association*" OR "joint exposure*" OR "overall effect*" OR "overall association*" OR "overall exposure*" OR "joint and individual" OR "co-pollutant*" OR "co-exposure*" OR "combined metal*" OR "mixed metal*" OR "principal component analysis" OR "exploratory factor analysis" OR "Bayesian Kernel Machine Regression" OR "Least Absolute Shrinkage and Selection Operator" OR "penalized regression*" OR "Weighted Quantile Sum" OR "quantile-based g-computation" OR	1,204,124

	"quantile g-computation" OR "cluster analysis" OR "Bayesian" OR "classification and regression tree*" OR "random forest*" OR "shrinkage-based" OR "shrinkage method*" OR "variable selection regression*" OR "structural equation model*" OR "multivariate adaptive regression spline*" OR "exposure surface smoothing" OR "novel approach and least-angle regression" OR "machine learning" (Title) or "mixture*" OR "multi-pollutant*" OR "multiple metals" OR "multi-metal*" OR "joint effect*" OR "joint association*" OR "joint exposure*" OR "overall effect*" OR "overall association*" OR "overall exposure*" OR "joint and individual" OR "co-pollutant*" OR "co-exposure*" OR "combined metal*" OR "mixed metal*" OR "principal component analysis" OR "exploratory factor analysis" OR "Bayesian Kernel Machine Regression" OR "Least Absolute Shrinkage and Selection Operator" OR "penalized regression*" OR "Weighted Quantile Sum" OR "quantile-based g-computation" OR "quantile g-computation" OR "cluster analysis" OR "Bayesian" OR "classification and regression tree*" OR "random forest*" OR "shrinkage-based" OR "shrinkage method*" OR "variable selection regression*" OR "structural equation model*" OR "multivariate adaptive regression spline*" OR "exposure surface smoothing" OR "novel approach and least-angle regression" OR "machine learning" (Abstract) ǀ Timespan: 1998-01-01 to 2021-10-01 (Publication Date)	
6	#1 OR #2 ǀ Timespan: 1998-01-01 to 2021-10-01 (Publication Date)	4,863,674
7	#3 OR #4 ǀ Timespan: 1998-01-01 to 2021-10-01 (Publication Date)	1,136,396
8	#5 AND #6 AND #7 ǀ Timespan: 1998-01-01 to 2021-10-01 (Publication Date)	1433

Table S2. Metal concentrations from the studies included in this review

Source	Exposure matrix	Metal concentrations		
		**Median (IQR)**	**Mean (SD)**	**GM (GSD)**
			Blood pressure and hypertension	
Park et al. 2017	Blood and urine (µg/dL)			Sb: 0.06 (2.21)<br>Total As: 8.88 (3.17)<br>Arsenous acid: 0.65 (1.91)<br>Arsenic acid: 0.68 (1.21)<br>Arsenobetaine: 1.99 (5.19)<br>Arsenocholine: 0.30 (1.91)<br>Dimethylarsonic acid: 3.85 (2.27)<br>Monomethylarsonic acid: 0.73 (1.85)<br>Ba: 1.20 (2.72)<br>Cd: 0.24 (2.77)<br>Co: 0.34 (2.26)<br>Cs: 4.22 (2.00)<br>Pb: 0.51 (2.47)<br>Mo: 38.64 (2.42)<br>Tl: 0.14 (2.09)<br>W: 0.07 (2.77)<br>U: 0.01 (2.65)
Wang et al. 2018	Blood and urine (µg/L for all but serum Pb (µg/dL))			In blood<br>Cd: 0.37 (2.22)<br>Pb: 1.32 (2.00)<br>Total Hg: 0.93 (2.68)<br><br>In urine<br>Sb: 0.06 (2.21)<br>Total As: 8.85 (3.16)<br>Arsenobetaine: 1.98 (5.17)<br>monomethylarsonic acid: 0.73 (1.85)<br>dimethylarsinic acid: 3.84 (2.26)<br>Ba: 1.20 (2.72)<br>Cd: 0.24 (2.77)<br>Co: 0.34 (2.25)<br>Cs: 4.21 (2.01)

Source	Exposure matrix	Metal concentrations		
		**Median (IQR)**	**Mean (SD)**	**GM (GSD)**
				Pb: 0.50 (2.47)<br>Hg: 0.39 (2.93)<br>Mo: 38.59 (2.42)<br>Tl: 0.14 (2.09)<br>W: 0.07 (2.77)<br>U: 0.01 (2.64)
Kupsco et al. 2019	Blood (μg/dL)		As: 0.085 (0.058)<br>Cd: 0.029 (0.019)<br>Co: 0.022 (0.024)<br>Cr: 0.080 (0.128)<br>Cs: 0.310 (0.135)<br>Cu: 158 (32)<br>Mn: 1.5 (0.50)<br>Pb: 3.7 (2.7)<br>Sb: 0.38 (0.10)<br>Se: 25 (4.5)<br>Zn: 613 (130)	
Warembourg et al. 2019	Blood (μg/L)	As: 1.2 (0.3, 2.3)<br>Cd: 0.2 (0.1, 0.3)<br>Co: 0.2 (0.1, 0.3)<br>Cs: 1.6 (1.2, 2.1)<br>Cu: 1420 (1270, 1610)<br>Hg: 1.9 (1.0, 3.4)<br>Mn: 11.1 (8.5, 14.3)<br>Mo: 0.6 (0.5, 0.8)<br>Pb: 9.7 (7.1, 13.2)<br>Tl: 2.0 (2.0, 2.0)		
Castiello et al. 2020	Urine (μg/g, creatinine adjusted)			As: 12.1 (5.02, 25.7)[a]<br>Cd: 0.04 (0.03, 0.06)[a]<br>Hg: 0.30 (0.17, 0.57)[a]<br>Ni: - (<0.55, 3.03)[a]<br>Pb: 0.24 (0.16, 0.34)[a]<br>Cr: - (<0.44, 1.22)[a]<br>Mn: - (<0.02, 0.82)[a]

**Source**	**Exposure matrix**	**Metal concentrations**		
		**Median (IQR)**	**Mean (SD)**	**GM (GSD)**
Desai et al. 2021	Blood and urine (μg/dL for Pb; μg/L for Hg; and μg/g creatinine for As and Cd)	In blood<br>Pb: 0.57 (0.26, 1.60)[b]<br>Hg: 0.37 (0.19, 2.12)[b]<br><br>In urine<br>As: 5.61 (1.37, 33.2)[b]<br>Cd: 0.06 (0.03, 0.23)[b]		
Everson et al. 2021	Blood and urine (μg/g for Ba, Cd, Co, Cs, Mo, Sb, Tl, and W; μg/dL for Pb)	In blood<br>Pb: 1.5 (1, 2.2)[c]<br><br>In urine<br>Ba: 1.33 (0.75, 2.31)[c]<br>Cd: 0.23 (0.13, 0.40)[c]<br>Co: 0.28 (0.19, 0.42)[c]<br>Cs: 4.24 (3.23, 5.67)[c]<br>Mo: 37.3 (25.4, 52.4)[c]<br>Sb: 0.10 (0.06, 0.15)[c]<br>Tl: 0.15 (0.11, 0.20)[c]<br>W: 0.06 (0.03, 0.11)[c]		
Howe et al. 2021	Urine (μg/L for Co, Se, Mo, As, Cd, Sb, and Pb, and mg/L for Mg)	Mg: 71.3 (50.5, 100.9)[d]<br>Co: 0.46 (0.28, 1.01)[d]<br>Se: 21.8 (17.3, 27.0)[d]<br>Mo: 64.9 (50.5, 86.7)[d]<br>As: 12.2 (5.2, 34.5)[d]<br>Cd: 0.47 (0.30, 0.69)[d]<br>Sb: 0.05 (0.04, 0.07)[d]<br>Pb: 1.00 (0.69, 1.48)[d]		Mg: 66.9 (61.3, 72.9)[d]<br>Co: 0.54 (0.48, 0.61)[d]<br>Se: 21.72 (20.72, 22.77)[d]<br>Mo: 65.26 (61.13, 69.66)[d]<br>As: 15.21 (12.50, 18.49)[d]<br>Cd: 0.45 (0.41, 0.50)[d]<br>Sb: 0.05 (0.05, 0.06)[d]<br>Pb: 0.92 (0.82, 1.04)[d]
Kim and Park. 2021	Blood (μg/dL for Pb, and μg/L for Hg and Cd)			Pb (μg/dL): 1.89 (1.88, 1.91)[a]<br>Hg (μg/L): 3.35 (3.31, 3.39)[a]<br>Cd (μg/L): 0.94 (0.93, 0.95)[a]
Shih et al. 2021	Toenail (μg/g)	Al: 401.89 (342.89)<br>V: 0.99 (0.85)<br>Cr: 1.21 (1.11)<br>Mn: 14.71 (14.63)		

Source	Exposure matrix	Metal concentrations		
		**Median (IQR)**	**Mean (SD)**	**GM (GSD)**
		Fe: 753.62 (638.22)<br>Co: 0.35 (0.31)<br>Ni: 2.76 (2.29)<br>Cu: 4.91 (1.98)<br>Zn: 134.12 (41.80)<br>As: 1.69 (2.04)<br>Se: 0.69 (0.19)<br>Mo: 0.04 (0.03)<br>Cd: 0.11 (0.13)<br>Sn: 0.23 (0.22)<br>Hg: 0.19 (0.17)<br>Pb:1.90 (1.66)<br>U: 0.07 (0.02)		
Xu et al. 2021	Blood (ng/mL)	Cd: 0.4 (0.2, 0.8)<br>Pb: 1.2 (0.8, 1.9)<br>Hg: 0.9 (0.5, 1.9)<br>Mn: 8.4 (6.9, 10.6)<br>Se: 198.0 (184.2, 215.6)		
Yao et al. 2021	Blood and urine (μg/dL)		In blood<br>Pb: 1.71 (1.66)<br>Cd: 0.52 (0.58)<br>Total Hg: 1.59 (2.45)<br><br>In urine<br>Total As: 20.49 (55.39)<br>Cd: 0.4 (0.47)<br>Pb: 0.78 (1.33)	
Zhang et al. 2021	Blood (μg/dL)	Pb: 2.42 (1.65, 3.68)<br>Hg: 2.15 (1.06, 3.70)<br>Cd: 0.69 (0.46, 1.04)<br>Se: 278.00 (248.00, 316.00)<br>Mn: 37.30 (28.80, 48.00)	Pb: 3.29 (3.03)<br>Hg: 3.15 (3.60)<br>Cd: 0.86 (0.68)<br>Se: 289.50 (60.49)<br>Mn: 39.57 (15.28)	
Zhong et al. 2021	Urine (μg/g creatinine)	As: 73.33 (55.46, 103.91)<br>B: 384.13 (263.95, 577.67)		

Source	Exposure matrix	Metal concentrations		
		**Median (IQR)**	**Mean (SD)**	**GM (GSD)**
		Ba: 5.37 (2.12, 11.10)<br>Bi: 16.92 (1.39, 45.61)<br>Cd: 2.35 (1.15, 5.22)<br>Co: 2.46 (0.22, 6.20)<br>Cr: 18.74 (6.66, 34.01)<br>Cu: 14.19 (9.07, 21.64)<br>Fe: 14.27 (4.76, 37.40)<br>Li: 13.55 (8.08, 20.84)<br>Mg: 32.49 (21.49, 49.64)<br>Mn: 0.39 (0.13, 1.43)<br>Mo: 73.71 (38.21, 133.05)<br>Rb: 308.52 (127.10, 555.63)<br>Se: 33.95 (7.95, 68.31)<br>Sr: 115.73 (69.33, 187.31)<br>Zn: 462.98 (281.81, 801.68)		
Zuk et al. 2021	Blood (μmol/L for Pb and Se; nmol/L for the others)		Female<br>Cd: 8.18 (2.82)<br>Hg: 15.79 (3.88)<br>Pb: 0.12 (2.93)<br>Se: 2.13 (1.16)<br><br>Male<br>Cd: 7.79 (3.11)<br>Hg: 21.40 (3.95)<br>Pb: 0.19 (2.53)<br>Se: 2.24 (1.14)	
Preeclampsia				
Bommarito et al. 2019[e]	3rd trimester urine (μg/L)	As: 17.9 (9.59, 32.6)[d]<br>Ba: 1.93 (0.98, 3.34)[d]<br>Cd: 0.08 (0.04, 0.14)[d]<br>Cu: 8.96 (6.73, 12.1)[d]<br>Hg: 0.51 (0.27, 0.97)[d]<br>Mn: 0.73 (0.51, 1.13)[d]<br>Mo: 51.3 (37.1, 68.8)[d]		

Source	Exposure matrix	Metal concentrations		
		Median (IQR)	Mean (SD)	GM (GSD)
		Ni: 2.84 (1.88, 3.97)[d]<br>Pb: 0.35 (0.15, 0.62)[d]<br>Se: 37.0 (29.6, 45.6)[d]<br>Sn: 0.63 (0.35, 1.22)[d]<br>Tl: 0.13 (0.08, 0.18)[d]<br>Zn: 242 (146, 364)[d]<br>Be, Cr, U, and W: Metals with >70% of samples below the LOD		
Wang et al. 2020	Blood (µg/L for Cr, Co, Ni, As, Cd, Sb, and Hg, and µg/dL for Pb)	Cr: 8.52 (2.68, 33.52)<br>Co: 0.37 (0.05, 1.83)<br>Ni: 7.46 (1.50, 14.76)<br>As: 15.55 (8.39, 23.00)<br>Cd: 1.11 (0.61, 4.26)<br>Sb: 1.67 (0.55, 10.59)<br>Hg: 1.50 (0.96, 2.22)<br>Pb: 2.96 (2.25,3.84)		
Liu et al. 2021	Blood (µg/L)	Ba: 1.09 (0.80, 1.57)<br>Co: 0.26 (0.23, 0.31)<br>Cs: 0.39 (0.30, 0.49)<br>Cu: 1873.77 (1632.56, 2106.86)<br>Mo: 1.94 (1.36, 2.68)<br>Sb: 3.03 (2.51, 3.60)<br>Se: 122.88 (113.58, 133.02)<br>Zn: 803.34 (712.82, 898.97)		
Dyslipidemia and serum lipid levels				
Park et al. 2014	Blood and urine	NA	NA	NA
Kupsco et al. 2019	*Please see above*			
Zhu et al. 2020	Blood (µg/L)			Sr: 23.15 (18.27, 29.03)[f]<br>Cd: 1.06 (0.66, 1.58)[f]<br>Pb: 28.07 (20.35, 36.15)[f]<br>V: 0.73 (0.46, 1.02)[f]<br>Al: 67.94 (42.58, 115.85)[f]

Source	Exposure matrix	Metal concentrations		
		**Median (IQR)**	**Mean (SD)**	**GM (GSD)**
				Co: 0.14 (0.10, 0.19)[f]<br>Mn: 27.05 (24.15, 29.49)[f]
Jiang et al. 2021	Blood (μg/L)	Al: 47.20 (29.14, 92.27)<br>Sb: 0.12 (0.07, 0.20)<br>As: 1.86 (1.21, 3.42)<br>Ba: 32.82 (21.94, 60.39)<br>Co: 0.16 (0.12, 0.20)<br>Cu: 951.51 (841.44, 1068.77)<br>Pb: 12.73 (8.74, 20.09)<br>Mn: 3.71 (2.70, 5.23)<br>Mo: 1.32 (1.05, 1.70)<br>Ni: 2.72 (2.01, 4.02)<br>Rb: 348.71 (311.66, 391.10)<br>Se: 64.88 (56.03, 75.06)<br>Sr: 34.94 (29.61, 41.30)<br>Tl: 0.13 (0.10, 0.17)<br>Ti: 32.40 (25.54, 41.08)<br>V: 0.75 (0.56, 1.25)<br>Zn: 1157.38 (978.94, 2459.94)		Al: 57.29 (29.14, 92.27)[a]<br>Sb: 0.11 (0.07, 0.20)[a]<br>As: 2.05 (1.21, 3.42)[a]<br>Ba: 37.33 (21.94, 60.39)[a]<br>Co: 0.16 (0.12, 0.20)[a]<br>Cu: 943.35 (841.44, 1068.77)[a]<br>Pb: 15.14 (8.74, 20.09)[a]<br>Mn: 3.75 (2.70, 5.23)[a]<br>Mo: 1.33 (1.05, 1.70)[a]<br>Ni: 3.08 (2.01, 4.02)[a]<br>Rb: 349.65 (311.66, 391.10)[a]<br>Se: 65.26 (56.03, 75.06)[a]<br>Sr: 35.22 (29.61, 41.30)[a]<br>Tl: 0.13 (0.10, 0.17)[a]<br>Ti: 32.55 (25.54, 41.08)[a]<br>V: 0.87 (0.56, 1.25)[a]<br>Zn: 1752.26 (978.94, 2459.94)[a]
Li et al. 2021[g]	Blood and urine (μg/L)	In blood, Shimen area<br>Fe: 1014.72(733.75,1293.04)<br>Cu: 851.85(716.45,1019.25)<br>Zn: 907.25(802.21,1036.03)<br>Se: 111.28(93.80,136.68)<br><br>In urine, Shimen area<br>Al: 7.92(4.79,11.98)<br>Ti: 170.23(113.23,257.18)<br>V: 1.31(1.02,1.62)<br>Cr: 1.82(1.15,3.52)<br>Mn: 0.32(0.19,0.51)<br>Co: 0.32(0.21,0.51)<br>Ni: 2.70(1.59,4.82)<br>As: 57.67(41.22,83.51)		

Source	Exposure matrix	Metal concentrations		
		**Median (IQR)**	**Mean (SD)**	**GM (GSD)**
		Rb: 2324.83(1545.34,3209.57)		
		Sr: 149.35(95.07,229.90)		
		Mo: 88.47(58.98,138.67)		
		Cd: 2.70(1.48,4.79)		
		Sn: 0.26(0.18,0.41)		
		Sb: 0.08(0.06,0.13)		
		Ba: 1.89(1.04,3.43)		
		W: 0.07(0.04,0.12)		
		Tl: 0.81(0.53,1.19)		
		U: 0.01(0.01,0.02)		
		In blood, Huayuan area		
		Fe: 845.60(608.30,1129.81)		
		Cu: 801.60(680.36,1030.70)		
		Zn: 825.79(715.38,938.90)		
		Se: 65.78(55.81,76.36)		
		In urine, Huayuan area		
		Al: 8.73(5.21,14.70)		
		Ti: 77.01(48.27,124.82)		
		V: 0.72(0.53,0.94)		
		Cr: 1.12(0.67,1.62)		
		Mn: 0.35(0.18,1.04)		
		Co: 0.32(0.21,0.53)		
		Ni: 1.81(1.05,3.02)		
		As: 40.08(24.16,59.46)		
		Rb: 2365.91(1500.01,3434.93)		
		Sr: 93.72(51.43,157.02)		
		Mo: 111.02(66.38,185.67)		
		Cd: 2.15(1.20,3.54)		
		Sn: 0.26(0.16,0.44)		
		Sb: 0.09(0.06,0.13)		
		Ba: 1.41(0.68,2.71)		
		W: 0.07(0.04,0.12)		

**Source**	**Exposure matrix**	**Metal concentrations**		
		**Median (IQR)**	**Mean (SD)**	**GM (GSD)**
		Tl: 0.52(0.31,0.83)<br>U: 0.01(0.01,0.02)		
		CVD outcomes		
Domingo-Rel-loso et al. 2019	Urine (µg/g cre-atinine)	Co: 0.23 (0.13, 0.48)<br>Cu: 6.06 (3.78, 9.74)<br>Mo: 25.37 (13.41, 50.32)<br>Zn: 183.16 (95.85, 341.73)<br>Sb: 0.08 (0.03, 0.16)<br>Ba: 58.44 (31.71, 103.61)<br>Cd: 0.38 (0.23, 0.64)<br>Cr: 3.58 (2.27, 5.88)<br>V: 2.12 (1.32, 3.47)		
Kupsco et al. 2019			*Please see above*	
Liberda et al. 2019	Blood (µmol/L)		As: 0.07 (0.08)<br>Cd: 14.18 (14.22)<br>Co: 4.28 (10.05)<br>Cu: 15.27 (2.42)<br>Pb: 0.23 (0.25)<br>Hg: 34.15 (62.25)<br>Mo: 6.15 (3.12)<br>Ni: 21.84 (10.03)<br>Se: 2.19 (0.32)<br>Zn: 96.99 (12.24)	
Wen et al. 2019[e]	Blood (µg/dL)	Al: 25.98 (17.76, 41.78)<br>As: 1.18 (0.66, 2.39)<br>Cd: 0.05 (0.03, 0.08)<br>Co: 0.24 (0.19, 0.30)<br>Cu: 890.43 (751.50, 1029.45)<br>Fe: 1759.33 (1366.16, 2280.28)<br>Mn: 9.61 (7.61, 11.85)<br>Mo: 1.12 (0.90, 1.44)<br>Se: 97.42 (84.66, 113.42)<br>Tl: 0.11 (0.09, 0.15)		

Source	Exposure matrix	Metal concentrations		
		**Median (IQR)**	**Mean (SD)**	**GM (GSD)**
Xiao et al. 2019[e]	Blood (μg/dL)	Zn: 1033.04 (857.54, 1228.16)<br>Controls matched to ischemic stroke cases<br>Al: 56.78 (36.96, 103.64)<br>As: 1.86 (1.09, 3.77)<br>Ba: 38.33 (23.04, 67.81)<br>Co: 0.17 (0.14, 0.21)<br>Cu: 953.14 (850.30, 1071.60)<br>Pb: 13.30 (8.75, 23.75)<br>Mn: 2.79 (2.12, 3.90)<br>Hg: 0.55 (0.36, 0.84)<br>Mo: 1.27 (1.00, 1.66)<br>Ni: 2.44 (1.83, 3.55)<br>Rb: 348.50 (309.66, 394.25)<br>Se: 66.36 (57.50, 78.59)<br>Sr: 33.92 (28.56, 41.63)<br>Tl: 0.10 (0.08, 0.13)<br>Ti: 49.09 (41.49, 56.95)<br>W: 0.05 (0.03, 0.07)<br>V: 1.41 (1.13, 1.95)<br>Zn: 1383.21 (1046.25, 2775.78)<br><br>Controls matched to hemorrhagic stroke cases<br>Al: 60.23 (38.98, 131.83)<br>As: 1.91 (1.04, 4.38)<br>Ba: 41.37 (23.12, 69.01)<br>Co: 0.16 (0.13, 0.21)<br>Cu: 999.38 (884.52, 1120.40)<br>Pb: 13.18 (8.79, 23.70)<br>Mn: 2.97 (2.06, 4.05)<br>Hg: 0.55 (0.33, 0.86)<br>Mo: 1.34 (1.02, 1.71)<br>Ni: 2.36 (1.77, 3.36)<br>Rb: 357.90 (318.82, 396.32)		

Source	Exposure matrix	Metal concentrations		
		**Median (IQR)**	**Mean (SD)**	**GM (GSD)**
		Se: 68.86 (57.14, 78.66)		
		Sr: 34.59 (28.30, 40.55)		
		Tl: 0.10 (0.08, 0.14)		
		Ti: 44.13 (35.50, 55.49)		
		W: 0.05 (0.03, 0.07)		
		V: 1.42 (1.14, 2.01)		
		Zn: 1461.55 (1072.69, 2830.71)		
Cabral et al. 2021	Blood (μg/L for Mn, Fe, Cu, Zn, I, and Se; mg/L for SELENOP; and nM for free-Zn)	Mn: 1.04 (1.14)		
		Fe: 928 (433)		
		Cu: 1021 (333)		
		Zn: 728 (185)		
		I: 56.7 (15.9)		
		Se: 80.0 (19.1)		
		SELENOP: 5.3 (1.8)		
		Free-Zn: 0.59 (0.31)		
Liu et al. 2021	Blood (μg/L)	Cr: 1.352 (0.886, 1.902)		
		Mn: 12.219 (9.005 (15.767)		
		Co: 0.336 (0.268, 0.395)		
		Ni: 0.729 (0.311, 1.163)		
		Cu: 508.271 (401.244, 672.552)		
		Mo: 0.485 (0.256, 0.757)		
		Cs: 1.047 (0.779, 1.314)		
		Ba: 3.191 (1.994, 4.529)		
		Tl: 0.027 (0.019, 0.038)		
		Pb: 0.625 (0.269, 1.127)		
Yang et al. 2021	Urine (μg/mmol creatinine)	Al: 0.64 (0.34, 1.45)		
		Ti: 9.94 (6.82, 14.48)		
		V: 0.06 (0.04, 0.09)		
		Cr: 0.04 (0.03, 0.06)		
		Mn: 0.03 (0.01, 0.06)		
		Fe: 0.78 (0.50, 1.48)		
		Co: 0.03 (0.02, 0.05)		
		Ni: 0.37 (0.25, 0.63)		
		Cu: 0.91 (0.71, 1.22)		

Source	Exposure matrix	Metal concentrations		
		**Median (IQR)**	**Mean (SD)**	**GM (GSD)**
		Zn: 35.23 (20.58, 52.00)<br>As: 3.05 (2.05, 4.65)<br>Se: 1.63 (1.25, 2.12)<br>Rb: 152.76 (108.30, 207.83)<br>Sr: 13.17 (9.11, 23.48)<br>Mo: 5.51 (3.92, 8.37)<br>Cd: 0.08 (0.05, 0.18)<br>Sn: 0.04 (0.02, 0.05)<br>Sb: 0.01 (0.01, 0.02)<br>Ba: 0.18 (0.09, 0.37)<br>Tl: 0.03 (0.02, 0.04)<br>Pb: 0.11 (0.07, 0.16)<br>U: 0.002 (0.000, 0.003)		

Al, Aluminum; As, Arsenic; B, Boron; Ba, Barium; Be, Beryllium; Bi, Bismuth; Cd, Cadmium; Co, Cobalt; Cr, Chromium; Cs, Cesium; Cu, Copper; Fe, Iron; GM, geometric mean; GSD, geometric standard deviation; Hg, Mercury; I, Iodine; IQR, interquartile range; Li, Lithium; Mg, Magnesium; Mn, Manganese; Mo, Molybdenum; Ni, Nickel; Pb, Lead; Rb, Rubidium; Sb, Antimony; SD, standard deviation; Se, Selenium; Selenop, selenoprotein; Sn, Tin; Sr, Strontium; Ti, Titanium; Tl, Thallium; U, Uranium; V, Vanadium; W, Tungsten; Zn, Zinc; [a] GM (IQR); [b] The range between 5th and 95th percentiles; [c] Weighted median; [d] Specific gravity-adjusted median or geometric mean; [e] Among non-cases; [f] GM (95% confidence interval); [g] The range of the summary measure was not specified to be as accurate as possible (e.g., IQR or 5th and 95th percentile range).

Table S3. Comparisons of the mixtures analysis methods most commonly used in this review

**Principal component analysis (PCA)**	**Bayesian kernel machine regression (BKMR)**	**Weighted quantile sum regression (WQSR)**
Unsupervised	Supervised	
▪ Can be used to identify exposure patterns	▪ Can estimate joint association for the mixture (WQSR can estimate a single coefficient)	
▪ Relies on an orthogonal solution	▪ Can estimate individual chemical associations with the outcome (posterior inclusion probabilities from BKMR and weight from WQSR indicate each component's relative importance within a mixture)	
▪ Potentially driven by variables irrelevant to the outcomes	▪ Can be used for variable selection	
▪ Number of components to retain is determined by the investigator	▪ Semi-parametric	▪ Parametric
	▪ Nonadditivity	▪ Interactions between the mixture components need to be hard-coded
	▪ Nonlinearity	▪ Assumes a linear association between the mixture and outcome
	▪ Flexible directionality	▪ Homogeneous directionality assumed
	▪ Computationally expensive	-
	-	▪ Uses quantiles of exposure and choice of number of quantiles is subjective
